# Thermo-induced physically crosslinked polypeptide-based block copolymer hydrogels for biomedical applications

**DOI:** 10.1093/rb/rbad039

**Published:** 2023-04-20

**Authors:** Dan Zhao, Yan Rong, Dong Li, Chaoliang He, Xuesi Chen

**Affiliations:** CAS Key Laboratory of Polymer Ecomaterials, Changchun Institute of Applied Chemistry, Chinese Academy of Sciences, Changchun 130022, China; College of Applied Chemistry and Engineering, University of Science and Technology of China, Hefei 230026, China; CAS Key Laboratory of Polymer Ecomaterials, Changchun Institute of Applied Chemistry, Chinese Academy of Sciences, Changchun 130022, China; CAS Key Laboratory of Polymer Ecomaterials, Changchun Institute of Applied Chemistry, Chinese Academy of Sciences, Changchun 130022, China; College of Applied Chemistry and Engineering, University of Science and Technology of China, Hefei 230026, China; CAS Key Laboratory of Polymer Ecomaterials, Changchun Institute of Applied Chemistry, Chinese Academy of Sciences, Changchun 130022, China; College of Applied Chemistry and Engineering, University of Science and Technology of China, Hefei 230026, China; CAS Key Laboratory of Polymer Ecomaterials, Changchun Institute of Applied Chemistry, Chinese Academy of Sciences, Changchun 130022, China; College of Applied Chemistry and Engineering, University of Science and Technology of China, Hefei 230026, China

**Keywords:** injectable hydrogels, polypeptides, stimuli responsive, secondary conformation, tissue engineering, immunotherapy

## Abstract

Stimuli-responsive synthetic polypeptide-containing block copolymers have received considerable attention in recent years. Especially, unique thermo-induced sol–gel phase transitions were observed for elaborately-designed amphiphilic diblock copolypeptides and a range of poly(ethylene glycol) (PEG)-polypeptide block copolymers. The thermo-induced gelation mechanisms involve the evolution of secondary conformation, enhanced intramolecular interactions, as well as reduced hydration and increased chain entanglement of PEG blocks. The physical parameters, including polymer concentrations, sol–gel transition temperatures and storage moduli, were investigated. The polypeptide hydrogels exhibited good biocompatibility *in vitro* and *in vivo*, and displayed biodegradation periods ranging from 1 to 5 weeks. The unique thermo-induced sol–gel phase transitions offer the feasibility of minimal-invasive injection of the precursor aqueous solutions into body, followed by *in situ* hydrogel formation driven by physiological temperature. These advantages make polypeptide hydrogels interesting candidates for diverse biomedical applications, especially as injectable scaffolds for 3D cell culture and tissue regeneration as well as depots for local drug delivery. This review focuses on recent advances in the design and preparation of injectable, thermo-induced physically crosslinked polypeptide hydrogels. The influence of composition, secondary structure and chirality of polypeptide segments on the physical properties and biodegradation of the hydrogels are emphasized. Moreover, the studies on biomedical applications of the hydrogels are intensively discussed. Finally, the major challenges in the further development of polypeptide hydrogels for practical applications are proposed.

## Graphical abstract

**Figure:**
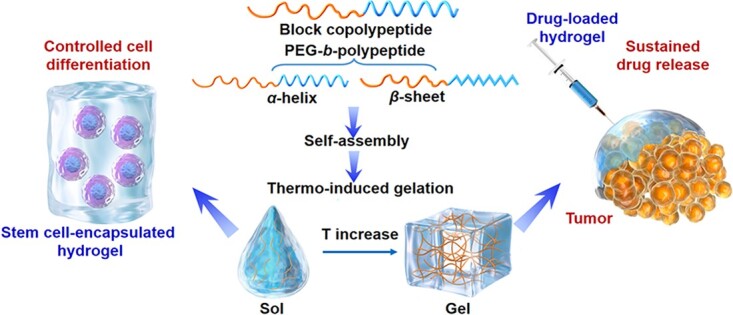

## Introduction

**Scheme 1.**
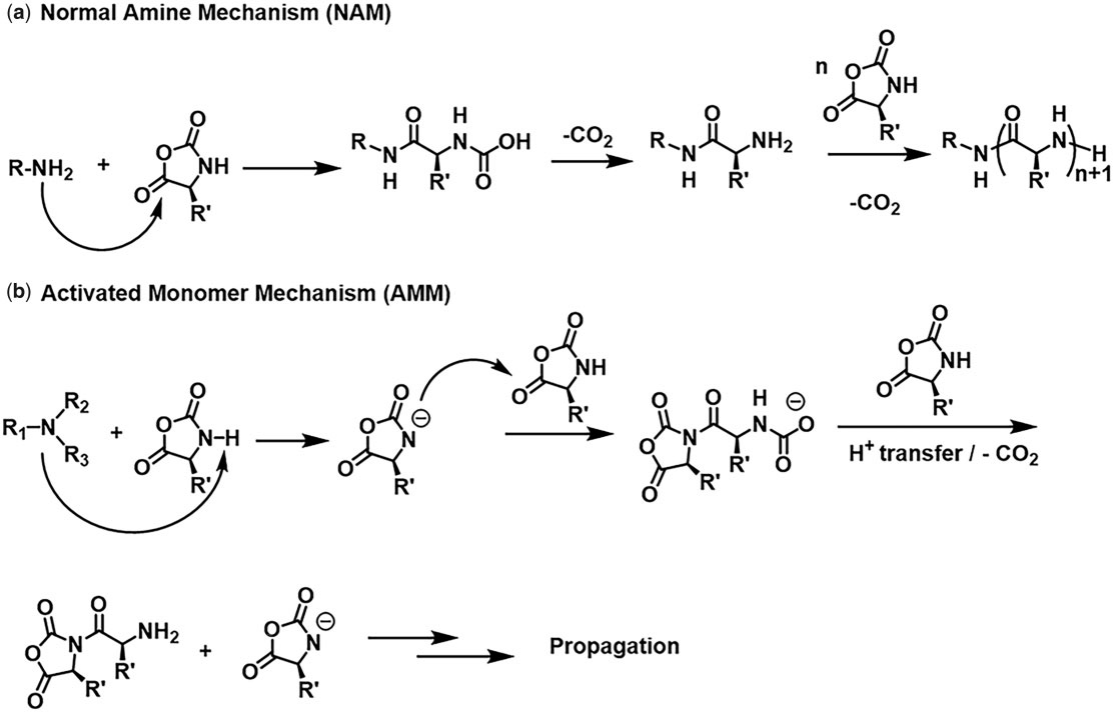
ROP of NCAs *via* (**a**) ‘normal amine mechanism’ and (**b**) ‘activated monomer mechanism’.

Synthetic polypeptides or poly(amino acid)s are a type of unique polymers comprising amino acid residues linked by peptide bonds [1]. Polypeptides have attracted progressive interest for biomedical applications in recent years, due to their biocompatibility, biodegradability as well as compositions and secondary structures analogical to natural proteins. Polypeptides are usually prepared by solid phase peptide synthesis, protein biosynthesis, or the ring-opening polymerization (ROP) of *α*-amino acid *N*-carboxyanhydrides (NCAs). Compared with other techniques, the synthesis approach through the ROP of NCA allows large scale production of polypeptides with high molecular weight (MW), and offers considerable chemical diversity of the products [2]. Two representative mechanisms for the ROP of NCAs are shown in Scheme 1 [3]. To control the synthesis of polypeptides *via* ROP of NCA in a controlled manner, various strategies have been developed. For instance, Co- or Ni-based complexes developed by Deming [4, 5] were used as initiators to prepare well-defined polypeptides and diblock copolypeptides. Subsequently, ammonium halides [6] and organosilicon initiators [7, 8] were reported to prepare polypeptides for preventing chain end termination. In addition, low temperature condition [9], high vacuum technique [10] and a nitrogen flow strategy [11] have been explored to prepare polypeptides with predictable MWs and narrow polydispersity index.

Hydrogels are 3D materials consisting of hydrophilic polymers and a significant amount of water. Due to their desirable physical characteristics and biocompatibility, hydrogels can be used as drug depots and cell culture scaffolds [12, 13]. In particular, polypeptide hydrogels have been widely developed and tested for biomedical applications recently, due to their good biodegradability, no acidic microenvironment during degradation and similarity to native extracellular matrix (ECM) [14]. In addition, injectable polypeptide hydrogels have attracted increasing attention, which are capable of gelation *in situ* after injection into body through a syringe or catheter triggered by physiologically relevant stimuli or the formation of covalent crosslinking bonds [15]. This type of material showed advantages in practical applications, such as minimal invasive implantation procedure and easy handling process. In addition to the formation of covalent crosslinking network by chemical reactions, the formation of physically crosslinked polypeptide hydrogels induced by physiologically relevant stimuli has attracted unique interest, due to their good biocompatibility without involving any chemical agents and the highly controlled manner of gelation process. Especially, thermo-induced gelation of the precursor polymer solutions may be achieved by simply adjusting the temperature from a lower temperature to the physiological temperature, which has advantages in the applications for *in situ* delivery of drugs, bioactive agents and cells. Accordingly, in the past two decades, the development of thermo-induced physically crosslinked polypeptide hydrogels has received intense interest, and their potential biomedical applications have been investigated.

**Scheme 2.**
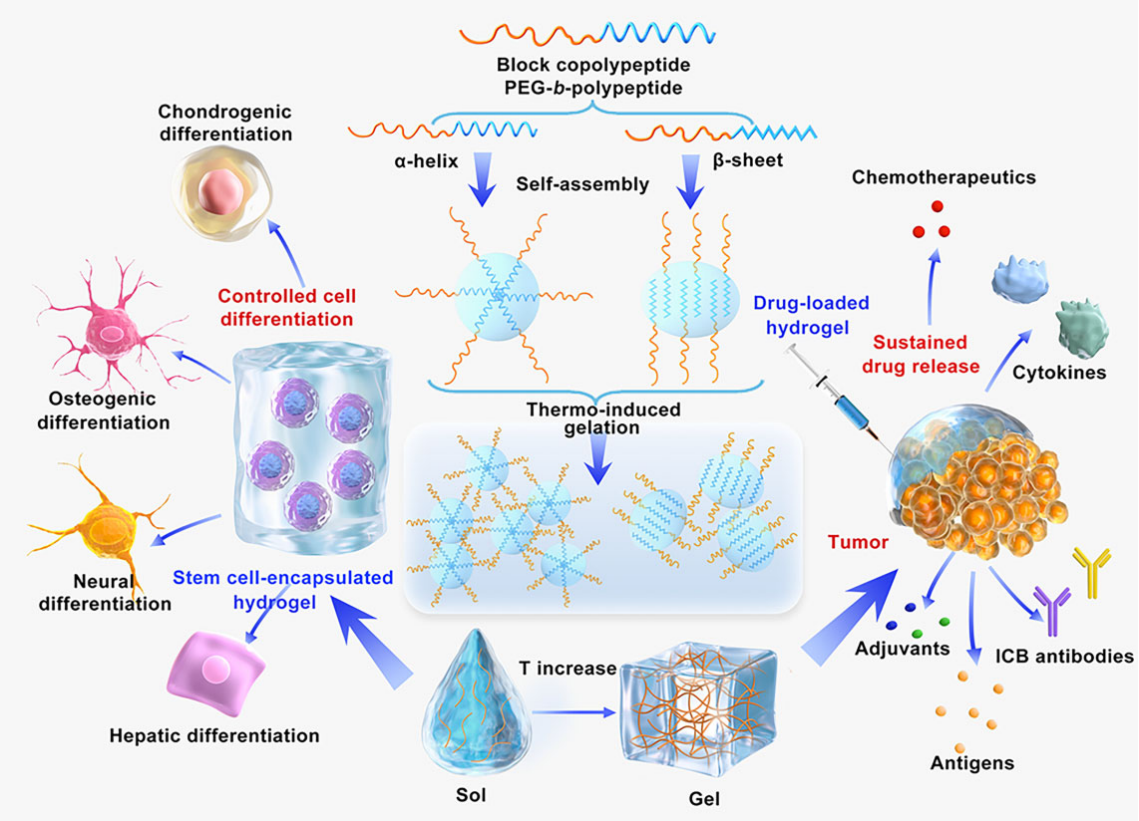
Schematic illustration for the mechanism of thermo-induced gelation of polypeptide-based block copolymers, and their potential applications for 3D cell culture and controlled cell differentiation, and for local sustained delivery of chemotherapeutics and/or immunostimulating agents.

In this review, the recent advances in the design and preparation of thermo-induced physically crosslinked polypeptide hydrogels are introduced, which are largely based on amphiphilic polypeptide-containing block copolymers including diblock copolypeptides with thermosensitive side chains and poly(ethylene glycol) (PEG)-*b*-polypeptide block copolymers. The stimuli-responsive gelation mechanisms and physical properties of the hydrogels are discussed. The potential biomedical applications of the polypeptide hydrogels, including scaffolds for 3D cell culture, depots for local delivery of antitumor agents and antibacterial materials, are emphasized (Scheme 2). In addition, some challenges for the further development of polypeptide hydrogels are proposed.

## Preparation and gelation mechanisms of physically crosslinked polypeptide hydrogels

**Scheme 3.**
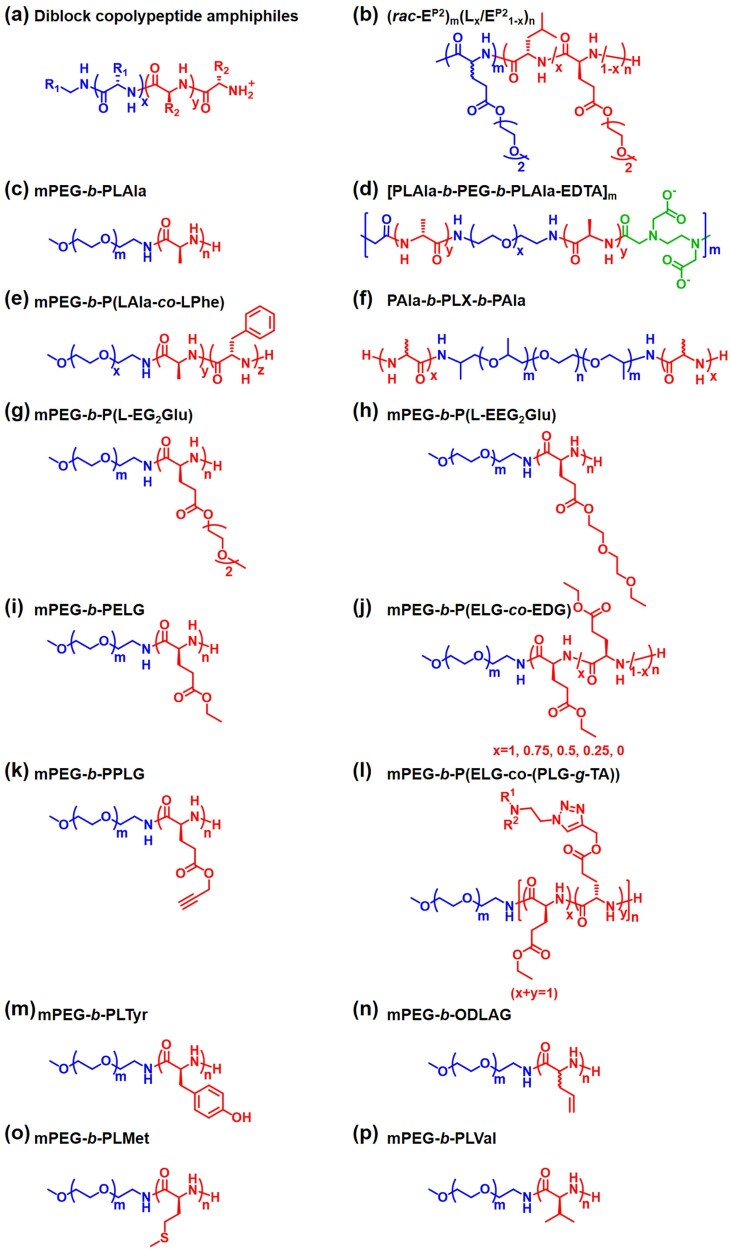
Chemical structures of some representative polypeptide-containing block copolymers. (a) Diblock copolypeptide amphiphiles. (b) (*rac*-EP^2^)_m_(L_x_/EP^2^_1-x_)_n_. (c) mPEG-*b*-PLAla. (d) [PLAla-*b*-PEG-*b*-PLAla-EDTA]_m_. (e) mPEG-*b*-P(LAla-*co*-LPhe). (f) PAla-*b*-PLX-*b*-PAla. (g) mPEG-*b*-P(L-EG_2_Glu). (h) mPEG-*b*-P(L-EEG_2_Glu). (i) mPEG-*b*-P(L-EEG_2_Glu). (j) mPEG-*b*-P(ELG-*co*-EDG). (k) mPEG-*b*-PPLG. (l) mPEG-*b*-P(ELG-*co*-(PLG-*g*-TA)). (m) mPEG-*b*-PLTyr. (n) mPEG-*b*-ODLAG. (o) mPEG-*b*-PLMet. (p) mPEG-*b*-PLVal.

Physically crosslinked hydrogels can be formed *in situ* through a sol–gel phase transition in response to external stimuli such as temperature, pH, redox, ion, light and enzyme [3]. Physical gelation is free from any chemical reaction since the formation of crosslinking networks is driven through various physical interactions including hydrogen bonds, hydrophobic interactions, electrostatic interactions, host-guest interactions, π–π stacking and so on [16]. Therefore, the crosslinking process is generally regarded to be biocompatible since potentially toxic chemical compounds and reactions are avoided [15]. It is noteworthy that the temperature-dependent sol–gel phase transitions have advantages in practical applications, since the bioactive agents can be mixed with the precursor solutions at a lower temperature and the hydrogel formation may be spatio-temporally controlled by simply changing the temperature. The chemical structures of typical polypeptide block copolymers are listed in Scheme 3, and their physical properties are listed in Table 1.

**Table 1.** Physical properties of typical thermo-gelling polypeptide-based block copolymer hydrogels

Polymer codes	Secondary structure^a^	T_gel_ (°C)^b^	C_gel_ (wt%)^c^	G′ (kPa)	*t*_deg_ ^d^	Ref.
(*rac*-E^P2^)_m_-(L_x_/$E_{1-x}^{P2}$)_n_	α-helix	30.6–37	2–4	0.1–1		[17]
mPEG-*b*-PLAla	β-sheet	10–40	3–14	∼1		[18]
mPEG-*b*-P(LAla-*co*-LPhe)	α-helix	7–26	4–9	∼1	∼15 days in rats	[19, 20]
mPEG-*b*-PELG	β-sheet	15–30	6–12	0.5–1	3––4 weeks in rats	[21]
mPEG-*b*-P(LTyr)_6_	β-sheet	25–50	0.25–3.0	0.2	7 days by chymotrypsin	[22]
ODLAG-*b*-PEG-*b*-ODLAG	β-sheet	80–95	1–18	2–10	100 h with enzymes	[23]
mPEG-*b*-PLMet	β-sheet	18–54	6–15	∼8	6 weeks in rats	[24]
mPEG-*b*-PLVal	β-sheet	—	10–20	∼2	27 days in mice	[25, 26]

a Secondary conformation.

b Gelation temperature.

c Gelation concentration.

d Degradation time.

### Block copolypeptide hydrogels

Molecular self-assembly process plays an important role in supramolecular structure formation, such as micelles, vesicles, nanoparticles and hydrogels [27]. The self-assembly process can be driven by various non-covalent interactions including hydrophobic interaction, hydrogen bonding, ionic interaction and guest–host interaction. The self-assembled ordered structures are usually stabilized in aqueous solutions by hydrophilic segments. Polypeptides are attractive building blocks for construction of supramolecular structures due to their unique secondary structures such as *α*-helix and *β*-sheet and their responsiveness to external stimuli including temperature and pH [1, 28, 29]. To construct stable physical networks in aqueous systems, amphiphilic polypeptide-containing block copolymers are the most studied building blocks for the formation of polypeptide hydrogels.

One kind of injectable hydrogels based on chemically synthesized polypeptides are amphiphilic block copolypeptide hydrogels. Deming and co-workers synthesized a type of diblock copolypeptide hydrogels (DCH), which are comprised of a hydrophilic polyelectrolyte block such as poly(L-lysine) (PLLys) or poly(L-glutamic acid) (PLGlu) and a hydrophobic block such as poly(L-leucine) (PLLeu), poly(L-valine) (PLVal) or poly(L-alanine) (PLAla) (Scheme 3a) [30]. It was believed that the secondary structures including *α*-helix and *β*-sheet promoted gel formation, which is superior to a random coil conformation. The critical gelation concentrations (CGCs) of the block copolypeptide hydrogels were as low as 0.25–1 wt%, and the storage moduli (G′) of the hydrogels ranged from <0.1 kPa to near 1 kPa, depending on the composition and relative block length. It was found that the block copolypeptides tended to assemble into twisted fibrils instead of spherical micelles like many other amphiphilic block copolymers, such as PEG-polyester block copolymers [31]. These block copolypeptide hydrogels can be injected through a syringe due to their shear-thinning property and re-assemble into hydrogels *in vivo* after injection [32].

Due to existence of ionized polypeptide segments, the second structure and gelation ability of block copolypeptide hydrogels can be affected by pH and ion strength. Tsitsilianis and co-workers synthesized a poly(L-alanine)-*b*-poly(L-glutamic acid)-*b*-poly(L-alanine) (PLAla-*b*-PLGlu-*b*-PLAla) triblock copolymer containing a central pH-responsive PLGlu block [33]. At low pH, the carboxyl groups in the PLGlu segment are not ionized and the copolymer forms *α*-helical plates which can further assemble into giant nanobelts as the concentration increases. In contrast, at high pH, the carboxyl groups are ionized and the copolymer turns into random coils and forms *β*-sheet nanostructures when ionic strength is increased, which can further assemble into twisted superfiber to form a hydrogel at elevated concentrations. The results indicate that hydrophobic association, secondary structure and electrostatic repulsion have great impact on gelation behaviors of the block copolypeptide. The fibril assemblies were also found in the hydrogel of a coil-sheet poly(L-lysine)-*b*-poly(L-threonine) (PLLys-*b*-PLThr) block copolypeptide [34], where the balance between the intermolecular hydrogen bonding interactions of sheet-like poly(L-threonine) (PLThr) segment and charge repulsion exerted by coil PLLys segment was critical for hydrogelation. At concentrations below CGC, block copolypeptides containing a PLLys-based polyelectrolyte block were found to be cytotoxic, whereas those containing a PLGlu-based segment showed low cytotoxic [35]. However, both the PLGlu- and PLLys-containing building blocks exhibited low cytotoxic in hydrogel status, likely attributed to the fact that the polypeptide blocks were embedded within the hydrogel networks. When DCHs of L-lysine and L-leucine (PLLys-*b*-PLLeu) are injected into the forebrain of mice, no obvious toxicity to the central nervous system (CNS) was detected *in vivo* [32].

In addition to hydrophilic–hydrophobic equilibrium derived from main chain, changes in hydrophilicity and hydrophobicity of side groups and terminal group can also affect the self-assembly behavior of polypeptides. For instance, Deming and co-workers developed a hydrogel of PLLys-*b*-poly(*o*-nitrobenzyloxycarbonyl-L-lysine) (PLLys-*b*-P(oNB-LLys)) block copolypeptide containing photolabile *o*-nitrobenzyloxycarbonyl (oNB) side group. The hydrogel displayed a photo-responsive gel-to-sol phase transition in response to UV irradiation, resulted from the hydrophobic-to-hydrophilic transition of the P(oNB-LLys) segment caused by full cleavage of the oNB groups [36]. This system may be used as a photolysis-controlled drug release system.

**Figure 1.**
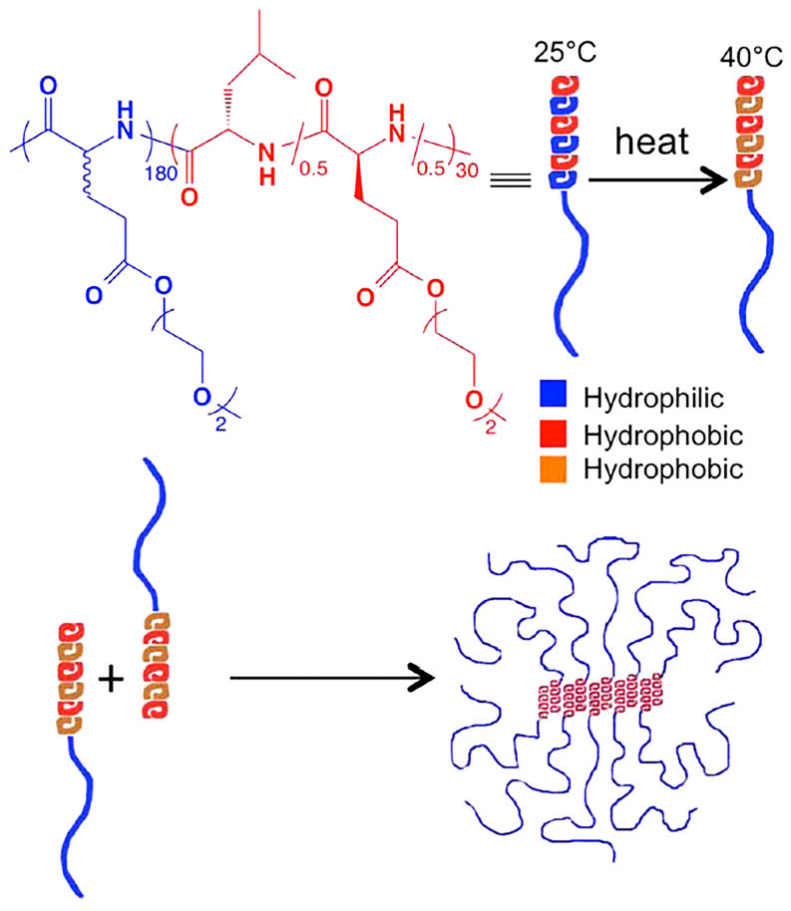
Schematic representation, structure and thermoresponsive gelation process for DCH_T_. Reproduced with permission from Ref. [17].

In another study of the same group, thermo-responsive oligo(ethylene oxide) (OEG) side-chain was incorporated into the side chain of poly(*rac*-glutamate) (PGlu) or PLGlu to fabricate a thermo-responsive nonionic block copolypeptide hydrogel (Fig. 1). The 3 wt% Phosphate buffered saline (PBS) solutions of poly(γ-(2-(2-methoxyethoxy)ethyl)-*rac*-glutamate)-*b*-poly(L-leucine-*co*-γ-(2-(2-methoxyethoxy)ethyl)-L-glutamate) block copolypeptide ((*rac*-E^P2^)_m_-(L_x_/$E_{1-x}^{P2}$)_n_, denoted as DCH_T_) showed thermo-induced sol–gel transitions at temperatures ranging from 30.6 to 37°C, dependent on the polymer composition. The 2–4 wt% copolypeptide hydrogels exhibited storage moduli (G′) at equilibrium of 0.1–1 kPa (Table 1). The block copolypeptides existed as a stable *α*-helix conformation with increasing temperature from 20 to 70°C. Thermo-induced reversible hydration-dehydration transition of OEG units is responsible for the reversible sol–gel transition (Scheme 3b) [17].

### Thermo-gelling PEG-polypeptide block copolymer hydrogels

PEG-containing amphiphilic block copolymers, especially PEG-polyester block copolymers, have been widely investigated for the potential thermo-gelling behaviors of their aqueous solutions at elevated concentrations [15, 29, 37, 38]. The mechanism for the thermo-gelling behaviors is related to the combined effects of the decline in the hydration effect of PEG blocks and the enhanced intermolecular interactions of the hydrophobic segments in response to temperature increase [39–41].

In recent years, PEG-polypeptide block copolymer hydrogels have also attracted increasing interest due to their good biocompatibility as well as unique intra- and inter-molecular interactions different from PEG-polyester block copolymers [1, 42]. The aqueous solutions of PEG-polypeptide block copolymers may be able to show a sol–gel phase transition in response to temperature increase, which can facilitate the injection of the polymer solutions through a syringe at a lower temperature and allow subsequent hydrogel formation *in situ* at an elevated temperature such as body temperature. To date, various PEG-polypeptide block copolymers have been synthesized *via* the ROP of *α*-amino acid NCAs containing hydrophobic or stimuli-responsive side chains, including L-alanine NCA, γ-substituted L-glutamate NCA, DL-allylglycine NCA, L-tyrosine NCA and OEGylated L-cysteine NCA, using amino-terminated PEG or methoxy PEG (mPEG) as a macroinitiator. The thermo-induced gelation behaviors and their potential biomedical applications of these block copolymers have been investigated.

#### Poly(ethylene glycol)-b-polyalanine and derivatives

**Figure 2.**
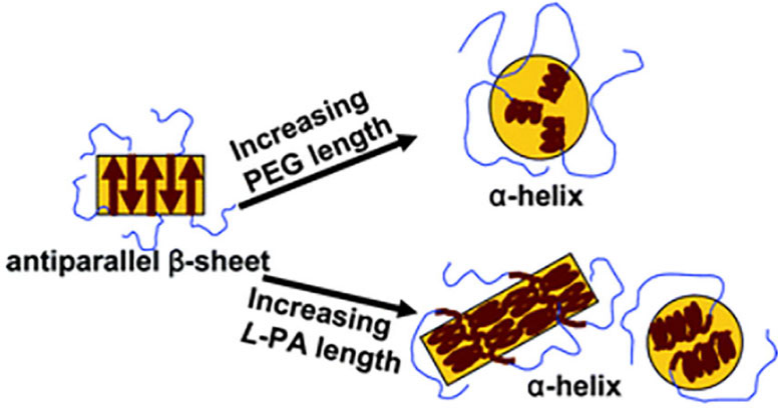
Variation of mPEG-L-PLAla self-assembly with the MW of each block. Reproduced with permission from Ref. [18].

Jeong and co-workers synthesized a methoxy-poly(ethylene glycol)-*b*-poly(L-alanine) diblock copolymer (mPEG-*b*-PLAla) (Scheme 3c), and the influence of the secondary structure of the polypeptide block on the gelation behavior of the diblock copolymer was investigated by comparison with mPEG-*b*-poly(DL-alanine) (mPEG-*b*-PAla) [43]. The diblock copolymers exhibited a heat-induced sol–gel phase transition in aqueous solutions. It was found that the formation of *β*-sheet conformation plays a critical role in developing a fibrous nanostructure and thermo-responsive hydrogel formation. The gelation temperatures of the 3–14 wt% mPEG-*b*-PLAla block copolymer aqueous solutions were in the range of 10–40°C. The maximum G′ of the hydrogels was ∼1 kPa. The *β*-sheet content increases with the augment of polymer concentration and temperature. Circular dichroism, Fourier transform infrared spectra and Cryogenic transmission electron microscopy showed that secondary structure of the PLAla block will change from antiparallel *β*-sheet to *α*-helical so as to decrease the steric hindrance comes from adjacent PEG segment, as the MW of PEG increased (Fig. 2) [18]. On the other hand, the MW increase of PLAla block can also facilitate the transformation of antiparallel *β*-sheet into *α*-helical structure. Correspondingly, the self-assembly structure changes from fibrous structure to spherical micelles. In addition, the block sequence [44], polymer backbone structure [45] and incorporation of ionic interactions [46] can also affect the secondary structure. The binding of Cu^2+^ to ethylene diamine tetraacetic acid-incorporated PEG/PLAla multiblock copolymer (Scheme 3d) can increase the *α*-helical content and facilitate thermogelation through effective salt-bridge formation. Utilizing star polymers can maximize hydrophobic interactions between adjacent alanine units compared with conventional diblock copolymers, thus decreasing the CGC as low as 3.5 wt% [47]. In an mPEG-*b*-PLAla-grafted chitosan system [48], the *α*-helical content can increase with the temperature rise or pH decline, which was proposed to be related to the hydrogen bonding between amino groups of chitosan and PEG. When a more hydrophobic amino acid, γ-benzyl-L-glutamate, was copolymerized with L-alanine, the *α*-helix structure content increased with the poly(γ-benzyl-L-glutamate) (PBLG) content [49]. The introduction of small number of PBLG unit can balance the *β*-sheet and *α*-helix conformation, thus increasing the modulus of hydrogel. However, when further increasing PBLG units, no obvious gel states were found. It was proposed that rigid helical structure hindered the sol-to-gel transition, while certain amount of random coil and *β*-sheet structures could facilitate hydrogel formation. From this point of view, the secondary structure of the polymer is influenced by many factors and the specific gelation mechanisms are also diversified. And the relationship between secondary structure and polymer structure as well as external environment cannot be summarized in a general rule. In addition, poly(*N*-vinyl pyrrolidone) can also be an alternative to PEG in developing polypeptide thermogels [50].

To adjust the gelation property, mPEG-*block*-poly(L-alanine-*co*-L-phenylalanine) (mPEG-*b*-P(LAla-*co*-LPhe)) thermogels were prepared (Scheme 3e) [51]. The polypeptide block copolymer existed as a typical *α*-helix conformation in aqueous solution. The 4–9 wt% mPEG-*b*-P(LAla-*co*-LPhe) aqueous solutions exhibited thermo-responsive sol–gel phase transitions at temperatures ranging from 7 to 26°C. The 5 wt% polypeptide hydrogel displayed a maximum G′ of ∼1 kPa. The polypeptides were stable in PBS solution, while they can be degraded in the presence of different enzymes *in vitro*. Following subcutaneous injection of the polypeptide hydrogels into rats, the hydrogels almost degraded in 15 days. Various proteolytic enzymes such as elastase, cathepsin B and cathepsin C may be responsible for the material degradation [19]. It was found by Hematoxylin and Eosin (H&E) staining that dense fibroblastic capsules formed surrounding the hydrogel and some host cells infiltrated into the polypeptide hydrogels, suggesting a mild inflammatory reaction. Additionally, it is expected that the degradation products of the hydrogel including amino acids and PEG may exhibit good biocompatibility without causing obvious pH change.

Jeong et al. reported that stereochemistry can also influence the degradation behavior and biocompatibility. Meanwhile, in contrast to the relatively rapid enzymatic degradation of mPEG-*b*-P(LAla-*co*-LPhe) hydrogels, the mPEG-*b*-poly(D-alanine-*co*-D-phenylalanine) (mPEG-*b*-P(DAla-*co*-DPhe)) hydrogels showed less degradation *in vivo*. In addition, H&E staining analysis showed that the mPEG-*b*-P(LAla-*co*-LPhe) hydrogel caused milder inflammation than the mPEG-*b*-P(DAla-*co*-DPhe) hydrogel [20]. Except for this, when partial L-alanine (LAla) units were replaced with D-alanine units, the secondary structure of mPEG-*b*-poly(alanine-*co*-phenylalanine) turned into random coils. The thermogelling temperature was observed at a much higher temperature and the size of nanoassemblies significantly decreased [52]. When mPEG-*b*-P(LAla-*co*-LPhe) was grafted to chitosan, 60–70% gel can be degraded in subcutaneous layer of rats in 1 week, which can be used as a short-term depots for pharmaceutical agents [53].

Compared with traditional poly(propylene glycol)-*b*-poly(ethylene glycol)-*b*-poly(propylene glycol) (Poloxamer or PLX), PAla end-capped PLX (PAla-*b*-PLX-*b*-PAla) (Scheme 3f) [54] showed prolonged gel duration from a few days to 1 month *in vivo*, depending on the chirality of alanine units. In addition, it was found that end-capping with hydrophobic groups may increase the *β*-sheet content and trigger the formation of nanofibrils [55], thus decrease the sol–gel transition temperature and increase the gel modulus. As the length of alky group in the chain end increased, the *β*-sheet conformation and the gel modulus also increased. Initial concentration can also affect gel modulus, nanofiber structure and cell viability [56]. When polymer concentration increases, the gel modulus, nanofiber population and thickness also increase. Jeong et al. reported a long-range nanofibrous orientation through the interaction of positively charged PAla-*b*-PLX-*b*-PAla and negatively charged hyaluronic acid (HA) [57]. The incorporation of HA resulted in the formation of biocompatible microenvironment for chondrocytes, which promoted marked cell clustering, increased cell proliferation and biomarker expression. Moreover, P(LAla-*co*-LPhe)-*b*-PLX-*b*-P(LAla-*co*-LPhe) block copolymers also exhibited the *β*-sheet increase and PLX dehydration as temperature increased [58], and the sol–gel transition temperature decreased with the increase of the P(LAla-*co*-LPhe) block length, decrease of PLX block length, or decrease of the ratio of Ala to Phe units.

Additionally, poly(LAla-*co*-L-leucine)-*b*-PLX-*b*-P(LAla-*co*-L-leucine) (P(LAla-*co*-LLeu)-*b*-PLX-*b*-P(LAla-*co*-LLeu)) was also synthesized to further adjust the physical property of the thermosensitive hydrogel. Compared with the PLAla-*b*-PLX-*b*-PLAla and P(LAla-*co*-LPhe)-*b*-PLX-*b*-P(LAla-*co*-LPhe) hydrogels, P(LAla-*co*-LLeu)-*b*-PLX-*b*-P(LAla-*co*-LLeu) could maintain *α*-helix structure even at temperatures above the sol–gel transition points, and the conformation change of PLX segment was believed to be the main driving force for the heat-induced sol–gel transition [59]. The hydrogel loaded with bovine serum albumin (BSA) exhibited a sustained BSA release profile without obvious initial burst for over 1 month *in vitro*. Additionally, the 10 wt% hydrogel injected in the subcutaneous layer of rats showed a duration of ∼47 days.

#### Thermo-responsive PEG-polyglutamate-based copolymer hydrogels

Thermo-responsive poly(glutamate) grafted with monomethyl-terminated oligo(ethylene glycol) (OEG) (P(EG_x_Glu), x represents the repeat number of ethylene glycol units in OEG chain) was reported by Li et al. [60], which is biodegradable compared with OEG-grafted poly(meth)acrylate. The reversible low critical solution temperature (LCST) behavior comes from the reversible hydration–dehydration transition of OEG side group. Furthermore, the studies showed that the increase of the OEG side chain led to the enhancement of the hydrophilicity and *α*-helix content of the polypeptide. The LCST can be tuned by varying the EG_2_Glu and EG_3_Glu ratio in the copolymers. However, poly(*rac*-EG_3_Glu) prepared by equal molar L-EG_3_Glu NCA and D-EG_3_Glu NCA do not show LCST behavior at temperature up to 70°C because the disruption of secondary structure from *α*-helix to random coil. In addition, LCST can also be tuned *via* random copolymerization of L-EG_x_Glu NCA with L-alanine NCA [61]. The LCST decreased when the hydrophobic L-alanine content increased. It was proposed that the order conformation plays a critical role in maintaining the LCST of P(L-EG_x_Glu), since the hydrogen bonding formation between polypeptide backbones can be enhanced, while the hydrogen bonding between polypeptide backbone and water will be diminished. Therefore, the *α*-helical formation was promoted and the LCST behavior of the polypeptide was affected. A series of mPEG-*b*-P(L-EG_2_Glu-*co*-LAla) diblock copolymers were subsequently prepared by the ROP of L-EG_x_Glu NCA and L-alanine NCA with amino-terminated mPEG as the macroinitiator. Instead of a thermo-induced sol–gel phase transition, the mPEG-*b*-P(L-EG_2_Glu-*co*-LAla) solution underwent a gel-sol transition and modulus decrease with increasing temperature.

**Figure 3.**
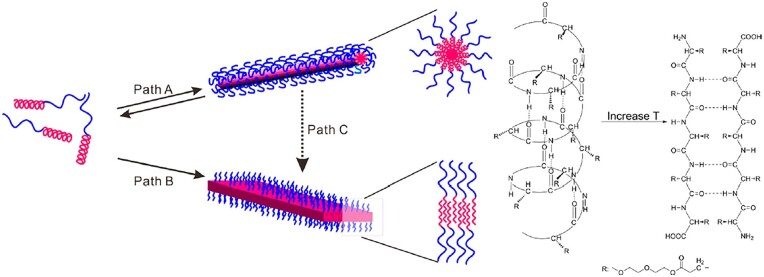
Two levels of self-assembly behavior of mPEG-*b*-P(L-EG_2_Glu). Reproduced with permission from Ref. [62].

In a separate study by the same group, the mPEG-*b*-P(L-EG_2_Glu) diblock copolymer was also synthesized *via* the ROP of L-EG_x_Glu NCA (Scheme 3g), which was found to show two stages of self-assembly behavior after increasing temperature followed by long-term thermal-annealing [62]. With increasing temperature at the first stage, the hydrophilic poly-L-EG_2_Glu block turns into hydrophobic block due to the dehydration of OEG side group. While the secondary structure kept an *α*-helical conformation in the heating process, leading to the formation of wormlike micelles (Fig. 3). In contrast, after thermal-annealing at 80°C for 12 h, the mPEG-*b*-P(L-EG_2_Glu) diblock copolymer exhibited *α*-helical to *β*-sheet transition, resulting in the formation of nanoribbons. It was assumed that the wormlike micelles were a metastable assembly during the formation of nanoribbons. Therefore, the long-term thermal-annealing promoted the transition from wormlike micelles to nanoribbons. In addition, it was found that the mPEG-*b*-P(L-EG_2_Glu) aqueous solutions spontaneously formed hydrogels at room temperature when the degree of polymerization (DP) of polypeptide was below 40 [63]. The alkyl-P(L-EG_2_Glu) amphiphilic hydrogels were also prepared by using alkyl amines as initiators [64]. The study showed that increase of alkyl length promoted the formation of intermolecular hydrogen bonding and thus led to the increase of *β*-sheet content. Correspondingly, the modulus of the hydrogel increased and the CGC of the polypeptide decreased.

In addition to the diblock copolymers, star-shaped P(L-EG_2_Glu) and P(L-EG_2_Glu)-*b*-PEG-*b*-P(L-EG_2_Glu) triblock copolymers were also developed for hydrogel formation. The star-shaped P(L-EG_2_Glu) synthesized by ROP of L-EG_2_Glu NCA using an amino-terminated dendrimer as the initiator adopted an *α*-helical conformation and was able to form hydrogels at low concentrations [65]. It was found that the CGC and hydrogel moduli were more dependent on arm numbers than arm length. For the ABA-type triblock copolymers, the longer P(L-EG_2_Glu) segment, the higher helical structure content [66]. In the triblock system, micellar packing mechanism was thought to be responsible for the hydrogelation, and the mechanical strength of hydrogel decreased when the temperature rose, due to the dehydration of polypeptide segments.

In addition to the change of polypeptide backbone, it was found that the subtle variation of the terminal group of OEG side chain also exhibited marked effect on the thermo-responsive gelation behaviors of the PEG-polyglutamate block copolymers. In a recent study by He and co-workers, diblock, triblock and four-armed star-shaped block copolymers composed of PEG and poly(γ-(2-(2-ethoxyethoxy)ethyl)-L-glutamate) (P(L-EEG_2_Glu)) were fabricated *via* the ROP of γ-(2-(2-ethoxyethoxy)ethyl)-L-glutamate NCA (L-EEG_2_Glu NCA) using amino-terminated mPEG, PEG or four-armed PEG as the initiators (Scheme 3h) [67]. It was reported that the increase of polypeptide block length of the PEG/P(L-EEG_2_Glu) block copolymers resulted in the enhancement of the *α*-helix content. A proper PEG/P(L-EEG_2_Glu) mass ratio of 1:1.5–2 facilitated the thermo-induced gelation. Notably, the subtle change of terminal group of the OEG side chain from methyl to ethyl group resulted in marked influence of the gelation property. It was observed the P(L-EEG_2_Glu)-*b*-PEG-*b*-P(L-EEG_2_Glu) containing ethyl-terminated OEG side groups showed lower heat-induced gelation concentration and lower gelation temperature at fixed polymer concentration, compared with P(L-EG_2_Glu)-*b*-PEG-*b*-P(L-EG_2_Glu) containing methyl-terminated OEG side groups. Moreover, the P(L-EEG_2_Glu)-*b*-PEG-*b*-P(L-EEG_2_Glu) hydrogel displayed more rapid thermoreversible sol–gel–sol transition with changing temperature between 37 and 0 °C, compared with the P(L-EG_2_Glu)-*b*-PEG-*b*-P(L-EG_2_Glu) hydrogel.

In addition, multiple stimuli-responsiveness can also be introduced into the OEG-modified polyglutamate hydrogels through the incorporation of pH-responsive L-glutamic acid units, reduction-responsive disulfide bond or redox-responsive diselenide bond [68–72].

Other than the OEG-modified polypeptide segments, thermo-responsive PEG-polypeptide hydrogels can also be developed by the incorporation of alkyl side groups into the polypeptide blocks. He, Chen and co-workers prepared mPEG-*block*-poly(γ-alkyl-L-glutamate) *via* the ROP of γ-alkyl-L-glutamate NCAs containing various alkyl groups using amino-terminated mPEG as the initiator [21]. It was found that the mPEG-*block*-poly(γ-ethyl-L-glutamate) (mPEG-*b*-PELG) (Scheme 3i) diblock copolymer showed markedly lower gelation temperature and higher storage moduli, compared with the diblock copolymer bearing methyl, n-propyl or butyl side groups. With increasing temperature, the 6–12 wt% mPEG-*b*-PELG solutions in PBS showed sol–gel phase transitions at temperatures ranging from 15 to 30°C. The 6 wt% polypeptide solutions exhibited a maximum G′ of 0.5–1 kPa with the temperature increase. It was proposed that the relatively high *β*-sheet content and appropriate hydrophilic/hydrophobic balance may be responsible for thermo-induced gelation of mPEG-*b*-PELG. This study also confirmed that the thermo-sensitive gelation behaviors can be affected by the subtle change of the substituent groups [73].

Besides the substituent groups, it was found that the manipulation of chirality of the amino acid residues also played a critical role in adjusting the gelation behaviors of the polypeptide hydrogels. In a recent study, He, Chen and co-workers synthesized mPEG-*block*-poly(γ-ethyl-glutamate) containing various γ-ethyl-L-glutamate (ELG) and γ-ethyl-D-glutamate (EDG) contents (Scheme 3j) [74]. It was found that the diblock copolymers containing mixed enantiomeric residues of ELG and EDG showed lower CGC and critical gelation temperature compared with mPEG-*b*-PELG or mPEG-*block*-poly(γ-ethyl-D-glutamate) (mPEG-*b*-PEDG). It was thought that the relatively high *β*-sheet conformation of diblock copolymer with mixed enantiomeric residues promoted the gelation. In a poly(D, L-alanine) hydrogel system, heat-induced increase of *β*-sheet structure was also observed [75].

Additionally, the mPEG-*b*-poly(γ-ethyl-glutamate) hydrogels showed no obvious degradation in PBS, whereas the gel degradation was accelerated in the presence of proteinases *in vitro*, due to the enzymatic cleavage of the peptide bonds [21, 74]. Moreover, the enzymatic degradation rate of the polypeptide hydrogels was found to be highly affected by the chirality of amino acid residues. With increasing the EDG content in the polypeptide segment, the degradation time was markedly prolonged to over 8 weeks following injection into the subcutaneous layer of rats, compared with 3–4 weeks for the mPEG-*b*-PELG hydrogels. Based on the histological analysis by H&E staining, augmented inflammatory response was seen in the skin tissue surrounding the hydrogel, indicating a mild foreign-body reaction elicited by the hydrogel. Following complete degradation of the mPEG-*b*-PELG hydrogel, the inflammatory reaction was found to vanish and no obvious tissue necrosis, hyperemia and edema were observed in the tissue. It was believed that the polypeptide hydrogels possess good biocompatibility. In addition, the inflammation was found to be enhanced with incorporation of D-amino acid residues in the polypeptide block.

Due to the existence of functional side groups of L-glutamate residues, the functionalization of the polypeptide hydrogels can be achieved through post-polymerization modification. In a study by Chen and co-workers, biofunctionalized thermo-responsive mPEG-polypeptide hydrogels were produced by incorporating azide-modified galactose and biotin moieties into mPEG-*b*-poly(γ-propargyl-L-glutamate) (mPEG-*b*-PPLG) (Scheme 3k) *via* click chemistry [76]. The galactose-modified polypeptide hydrogel was found to promote cell adhesion, probably due to the ability to adsorb fibronectin in cell-extracellular. In addition, a photo-responsive star-shaped four-armed PEG-b-poly(γ-*o*-nitrobenzyl-L-glutamate) copolymer hydrogel was prepared through the incorporation of *o*-nitrobenzyl ester in the polypeptide side group [77]. The hydrogel degradation can be triggered under UV irradiation, due to the hydrophobic-to-hydrophilic transition caused by the photolabile cleavage of o-nitrobenzyl ester group. The property can be used for photo-controlled drug release from the hydrogel.

**Figure 4.**
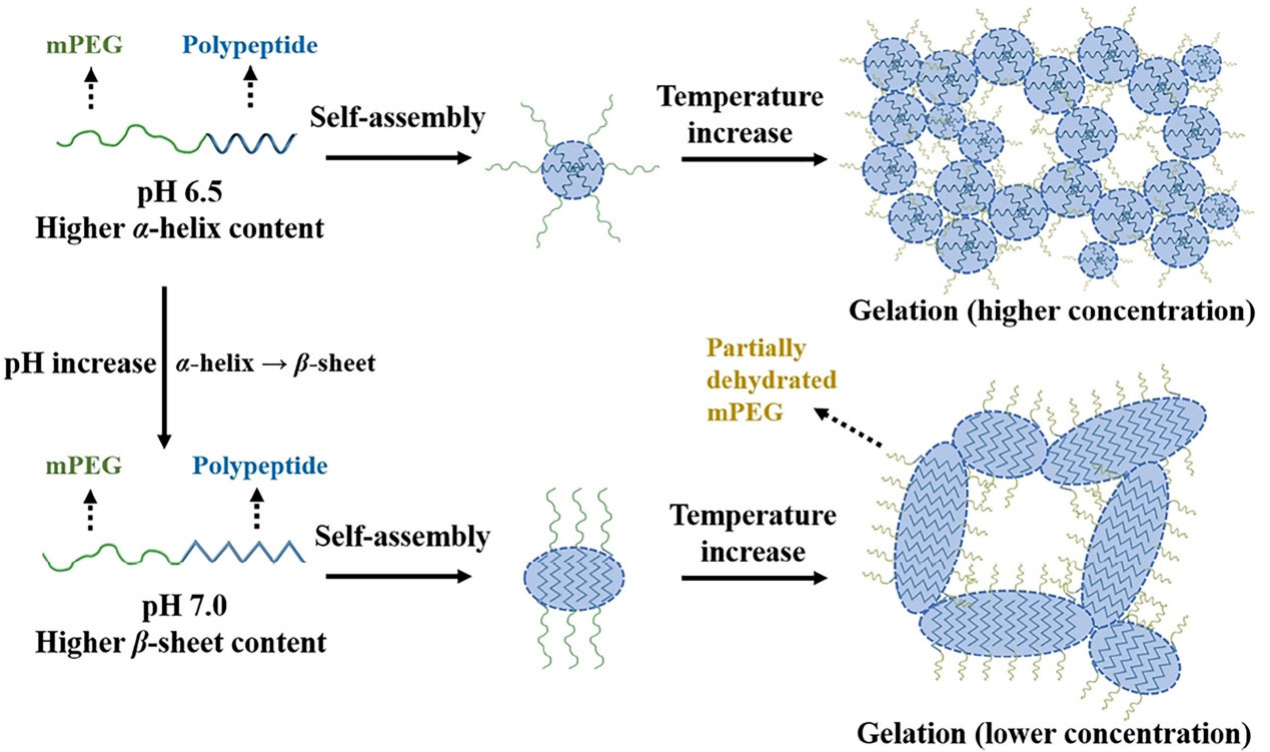
Schematic illustration of secondary structure transitions, self-assembly and thermo-induced gelation of mPEG-*b*-P(ELG-*co*-PLG-*g*-DEA) or (mPEG-*b*-P(ELG-*co*-PLG-*g*-PD) aqueous solutions at pH 6.5 and a neutral pH, respectively. Reproduced with permission from Ref. [78].

In recent studies by He and co-workers, a series of pH and temperature-responsive hydrogels were prepared by the ROP of ELG NCA and γ-propargyl-L-glutamate NCA (PPLG NCA) using mPEG-NH_2_ as the macroinitiator, followed by post-polymerization modification through the click chemistry between the propargyl groups and azido-conjugated tertiary amines (Scheme 3l) [78, 79]. It was found that the secondary conformation and thermo-induced sol–gel phase transitions of the mPEG-polypeptide block copolymers were dependent on the pH. For instance, 2-(diethylamino)ethyl- or 2-(1-piperidino)ethyl-modified mPEG-*b*-P(ELG-*co*-PLG) (mPEG-*b*-P(ELG-*co*-PLG-*g*-DEA) or (mPEG-*b*-P(ELG-*co*-PLG-*g*-PD)) predominantly adopted as a *α*-helix conformation at pH 6.5 but transformed into a structure with increased *β*-sheet content at a neutral pH. It was found that the increased *β*-sheet content in the polypeptide block led to marked reduction in the critical thermo-induced gelation concentration (Fig. 4).

#### PEG-poly(L-tyrosine) hydrogels

An mPEG-*block*-poly(L-tyrosine)_6_ (mPEG-*b*-P(LTyr)_6_) diblock copolymer was prepared by Heise and co-workers through the ROP of O-Benzyl-L-tyrosine NCA with amino-terminated mPEG as the initiator (Scheme 3m) [22]. The diblock copolymer aqueous solutions at a low concentration of 0.25–3.0 wt % showed heat-induced sol–gel transitions within 25–50°C. The 2 wt% polypeptide hydrogel showed a maximum G′ of ∼0.2 kPa with increasing temperature from 25 to 60°C. It was proposed that, with increasing temperature, the mobility of the PEG segment decreased, while the mobility of poly(L-tyrosine) (PLTyr) block increased, which promoted the polypeptide packing and self-assembly. It was found that the polypeptide hydrogel degraded completely in 7 days *in vitro* with the presence of chymotrypsin, due to its ability to cleave amide bonds adjacent to an aromatic amino acid.

On the other hand, in separate studies by Hamley and co-workers, no hydrogel was found for the aqueous solutions of PLTyr-*b*-PEG-*b*-PLTyr triblock copolymers with the DPs of each PLTyr block of 2 and 5, respectively [80, 81]. It is noteworthy that the incorporation of an aromatic Fmoc moiety to the end of P(LTyr)_2_-*b*-PEG-*b*-P(LTyr)_2_ triblock copolymer was able to self-assemble into *β*-sheet fibril-based hydrogels. The aromatic stacking interactions and the hydrogen bonds of backbone were assumed to be the driving forces for the self-assemble. And the bridging of PEG chains led to a self-supporting network.

It is noteworthy that the hydroxyl side groups of PLTyr segments can also be functionalized. Lu and co-workers synthesized a poly(*O*-diethylphospho L-tyrosine)_15_-*b*-PEG-*b*-poly(*O*-diethylphospho L-tyrosine)_15_ (P(pTyr)_15_-*b*-PEG-*b*-P(pTyr)_15_) triblock copolymer *via* the ROP of *O*-diethylphospho L-tyrosine NCA [82]. The triblock copolymer formed hydrogel in the presence of alkaline phosphatase (ALP), since ALP can induce the dephosphorylation of P(pTyr)_15_ blocks, thus promoting the physical aggregation of the partially dephosphorylated poly(L-tyrosine) blocks. It is suggested that the gelation property of the poly(L-tyrosine)-based triblock copolymer can be tuned by adjusting the hydrophilicity of the side group. Furthermore, due to the enzyme-responsiveness of tyrosine residues, the hydrogel can be strengthened by adding horseradish peroxidase and hydrogen peroxide, leading to the formation of intermolecular covalent crosslinking bonds between the tyrosine residues [82, 83].

#### PEG-poly(glycine) derivatives

Wooley and co-workers prepared a kind of thermo-responsive oligo(DL-allylglycine)-*b*-PEG-*b*-oligo(DL-allylglycine) (ODLAG-*b*-PEG-*b*-ODLAG) triblock copolymer *via* the ROP of DL-allylglycine with amino-terminated PEG as the initiator [23]. The 1–18 wt% triblock copolymer aqueous solutions underwent heat-induced sol–gel transition at a high-temperature range of 80–95°C. The *β*-sheet-driven fibril formation was proposed to be responsible for the physical gelation. The hydrogel showed relatively slow erosion for over 250 h in Tris–HCl buffer without enzyme, whereas the degradation was markedly accelerated to <100 h with addition of trypsin or proteinase K.

**Figure 5.**
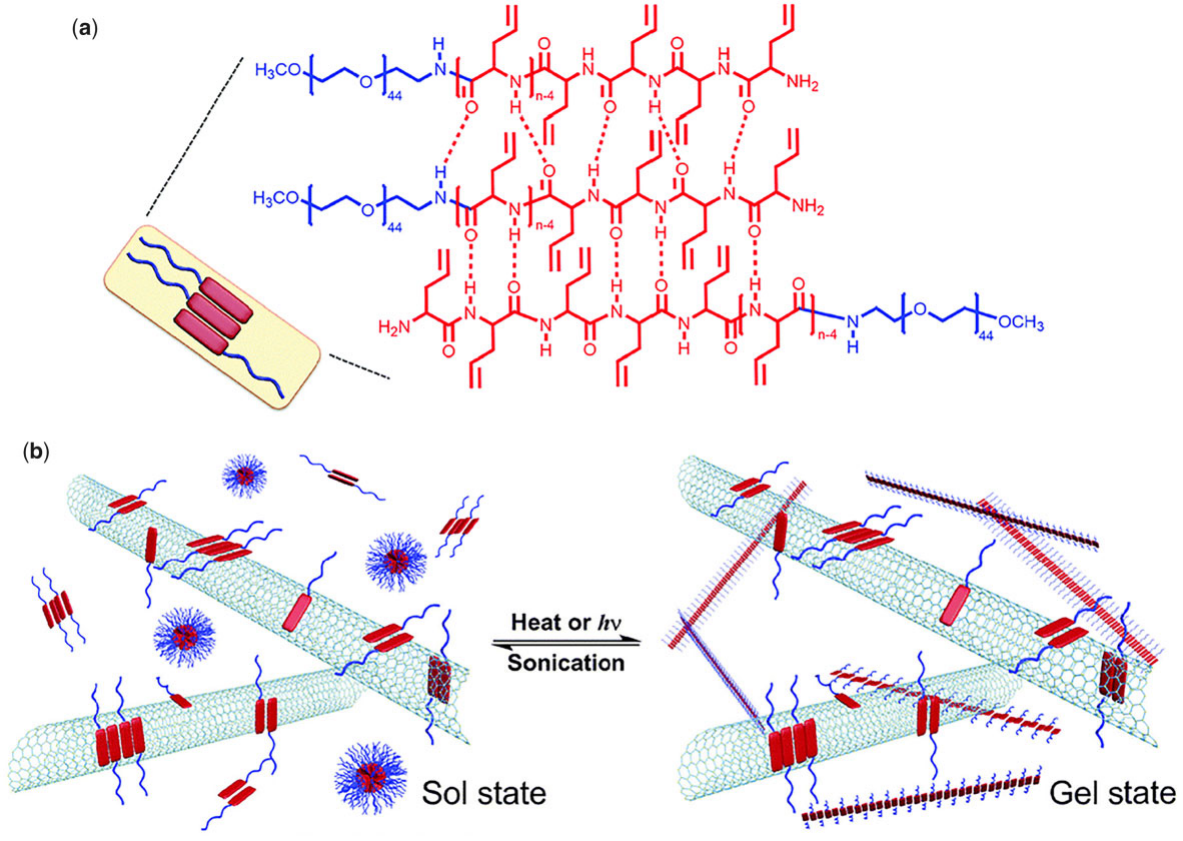
PEG-*b*-ODLAG/SWCNT hydrogel for reversible patterning of soft conductive materials. (**a**) Parallel or antipallel β-sheet conformation of nanofibrils. (**b**) Reversible conversion of polymeric supramolecular structures in response to stimulus. Reproduced with permission from Ref. [84].

To realize the controlled thermo-responsive gelation process, photo-thermally active single-walled carbon nanotubes (SWCNTs) were added into an mPEG-*b*-poly(DL-allylglycine) (PEG-*b*-ODLAG) (Scheme 3n) system by the same group [84]. Rapid photo-responsive sol–gel transition (<10 s) was achieved by converting the photo-thermal behavior of SWCNTs into the thermo-induced gelation of the polypeptide (Fig. 5). The material also showed good conductivity (>100 S m ^− 1^) and allowed for reversible photo-patterning.

#### Thermo-responsive PEG-poly(L-methionine) (PEG-PLMet) and derivatives

Some bioresponsive amino acids have also been incorporated into thermo-responsive polypeptide hydrogels. He, Chen and co-workers prepared an mPEG-*b*-PLMet diblock copolymer (Scheme 3o) *via* the ROP of L-methionine NCA [24]. The PLMet block with the DP of 10–20 adopted predominantly a *β*-sheet conformation, while the *α*-helix content increased with the DP increase. The 6–10 wt% copolymer solutions in PBS showed thermo-induced sol–gel transitions with the gelation temperatures ranging from 16 to 33°C. Moreover, the degradation of hydrogels could be triggered in the presence of physiologically relevant reactive oxygen species (ROS) such as H_2_O_2,_ attributed to the hydrophobic-to-hydrophilic transition of the polypeptide block caused by oxidation of methionine residues into hydrophilic sulfoxide and sulfone. The hydrogel degraded within 6 weeks *in vivo* after injection into the subcutaneous layer of rats. Model drug encapsulated within the hydrogel exhibited controlled release profiles in response to H_2_O_2_ concentration. Additionally, PEG-PMet also exhibited excellent cytoprotective ability under oxidative stress, indicating a potential as a scaffold for cell delivery under oxidative stress. In a subsequent study by Zhang and co-workers, a ROS-responsive four-armed PEG-PLMet hydrogel was synthesized through a similar ROP of L-methionine NCA using amino-terminated four-armed PEG as the initiator [85].

In addition to the ROS-responsive LMet residue, immunoactive amino acids such as 1-methyl-D-tryptophan (D-1MT) have also been introduced into the polypeptide segment. A ROS-responsive P(LMet-*co*-D-1MT)-*b*-PEG-*b*-P(LMet-*co*-D-1MT) triblock copolymer was synthesized through the copolymerization of LMet NCA and D-1MT NCA using amino-terminated PEG as the initiator [86]. The 4–10 wt% copolymer solutions in PBS exhibited heat-induced sol–gel transitions with gelation temperature of 12–30°C. The degradation of the polypeptide hydrogel lasted 5 weeks in the subcutaneous layer of rats. Since the co-existence of ROS-responsive LMet residues and immunoactive D-1MT, the polypeptide hydrogel was investigated as a depot for sustained delivery of immunotherapeutic agents.

#### Thermo-responsive PEG-b-poly(L-valine) (PEG-b-PLVal) and derivatives

Wang and co-workers prepared a kind of mPEG-*b*-poly(L-valine) (mPEG-*b*-PLVal) diblock copolymer (Scheme 3p) [25]. It was proposed that both *α*-helical and *β*-sheet existed in the polypeptide block with the DP of 4–19. The mPEG-*b*-PLVal hydrogel exhibited storage modulus ∼2 kPa [26]. Human fibroblast NIH/3T3 cells were cultured within the hydrogel as a 3D scaffold and the cell maintained a high viability. The cell proliferation study showed that metabolically active cells increased continuously during a culture period of 7 days, which demonstrated that the polypeptide hydrogel can promoted NIH/3T3 cell proliferation. The polypeptide hydrogel exhibited a degradation period of 27 days in the subcutaneous layer of C57BL/6J mice. Mild inflammation was observed at Days 3 and 10 post-implantation of the hydrogel, which was relieved after the degradation of the hydrogel, suggesting a good biocompatibility *in vivo*.

In a separate study by Liu and co-workers, a similar mPEG-*b*-PLVal diblock copolymer with the DP of polypeptide block of 2.8–11.5 was prepared [87]. It was reported that the PLVal block adopted a predominant *β*-sheet structure. The 2–8 wt% diblock copolymer solutions showed heat-induced sol–gel transitions with the transition temperatures ranging from 20 °C to 80 °C depending on the polymer concentration. It was assumed that the thermo-induced sol–gel transition of the polypeptide solutions was due to the aggregation of *β*-sheet fibers formed by the polypeptide segments and the heat-induced partial dehydration of PEG blocks.

Other than L-valine-based polypeptide, L-norvaline, an arginase 1 inhibitor, has also been used for fabrication of an immunoactive polypeptide hydrogels. Luan and co-workers prepared mPEG-*b*-poly(L-norvaline) (mPEG-*b*-PLNVal) diblock copolymer [88]. The diblock copolymer adopted a predominant β-sheet conformation. The 4–10 wt% aqueous solutions of mPEG-*b*-PLNVal showed heat-induced sol–gel transitions at temperatures ranging from 10 °C to 44 °C. The 6 wt% Dox-loaded hydrogel displayed a sustained drug release manner at pH 7.4 and 37°C over 6 days, which was accelerated by reducing pH or addition of proteinase K.

## Biomedical applications

### 3D cell culture and tissue engineering

Scaffolds for tissue engineering are designed and fabricated to adjust the physicochemical environment and biological clues of cells [89]. Hydrogels have been shown to be excellent platforms for 3D cell culture and tissue engineering [90, 91]. The 3D environments provided by hydrogels can affect cell morphology, mechano-responses, gene expression and cell proliferation and differentiation, which are quite different from 2D environment. Due to their good biocompatibility and porosity of hydrogels, various stem cells can be cultured and allowed to differentiate into specific kinds of cells through the addition of bioactive small molecules and proteins.

It has been established that the thermo-responsive, physically crosslinked polypeptide hydrogels possess excellent biocompatibility, adjustable degradation rates in the presence of enzymes and controlled sol–gel phase transitions. Thus, some polypeptide-based block copolymer hydrogels have been investigated as scaffolds for 3D culture of various stem cells including bone-marrow-derived mesenchymal stem cells (BMSCs), adipose-tissue-derived stem cells (ADSCs) and tonsil-derived mesenchymal stem cells (TMSCs) (Table 2).

**Table 2.** Typical applications of thermosensitive polypeptide hydrogels in 3D cell culture and tissue engineering

Sample codes	Cells in hydrogel	Bioactive agents	*In vivo* transplantation	Potential application	Ref.
mPEG-*b*-PLAla	ADSCs	—	BALB/c mice	Cartilage tissue engineering	[14]
	BMCAs	—	BALB/c mice		[92]
	Chondrocytes	—	—		[93]
mPEG-*b*-PLAla+GO/rGO	TMSCs	TGF-β3	—		[94]
mPEG-*b*-PLAla+functionlized PS microspheres	TMSCs	—	—		[95]
mPEG-*b*-PLAla-*b*-PLAsp + LDH	TMSCs	Kartogenin	—		[96]
PAla-*b*-PLX-*b*-PAla	Chondrocytes	—	Nude mice		[97]
mPEG-*b*-P(LAla-*co*-LPhe)	TMSCS	—	Osteochondral defect models of New Zealand white rabbits		[98]
mPEG-*b*-P(LAla-*co*-LPhe)+mesocrystals	TMSCs	—	—	Bone tissue engineering	[99]
mPEG-*b*-PLAla + MNP + HAP	MC3T3-E1 pre-osteoblasts	—	—		[100]
(rac-E^P2^)_m_(L_x_/$E_{1-x}^{P2}$)_n_	NSCs	bFGF	mGFAP-Cre-tdT reporter mice	Neural tissue engineering	[101]
mPEG-*b*-PLAla	TMSCs	BDNF and NGF	—		[102]
mPEG-*b*-PLAla	Fibroblasts	—	Rats	Skin tissue engineering	[103]
mPEG-*b*-PLAla	TMSCs	TUDCA, HGF and FGF4	—	Liver tissue engineering	[104]
LB-PLX-*b*-PLAla + PLX-*b*-PLAla	TMSCs	—	—		[105]

#### Cartilage tissue engineering

Injectable, thermo-responsive mPEG-*b*-PLAla hydrogels were investigated by Jeong and co-workers as a 3D matrix for culture of ADSCs [14]. ADSCs can be mixed with the polypeptide solutions at lower temperature, and cell-encapsulated hydrogels can be formed *in situ* by increasing temperature. After incubation for 14 days *in vitro*, the ADSCs within the hydrogel retained a spherical morphology and showed higher expression of type II collagen (Col II). Similarly, the ADSC-laden hydrogels exhibited predominant chondrogenic biomarkers following subcutaneous injection into BALB/c mice.

BMSCs were also cultured in the mPEG-*b*-PLAla thermo-responsive hydrogel [92]. It was found that BMSCs underwent dominant chondrogenesis in the polypeptide thermogel in the *in vitro* study. After implantation into the subcutaneous layer of BALB/c mice, the cells encapsulated in the hydrogel showed significant expressions of Col II and formation of sulfated glycosaminoglycan (sGAG), suggesting predominantly chondrogenic differentiation of BMSCs *in vivo*.

Due to their easy accessibility, higher population density and proliferation rate, TMSCs were also used for tissue engineering study [94]. A type of 2D/3D hybrid cell culture systems was constructed by suspending graphene oxide (GO) or reduced graphene oxide (rGO) into the mPEG-*b*-PLAla aqueous solution. The Col II expression increased in the 2D/3D hybrid system compared with the mPEG-*b*-PLAla 3D hydrogel without GO or rGO. The expressions of chondrogenic biomarkers such as Col II A1, SOX 9, Col X and Col II significantly increased in the GO/mPEG-*b*-PLAla hybrid hydrogel as compared with the mPEG-*b*-PLAla and rGO/mPEG-*b*-PLAla hydrogels, suggesting that the GO/mPEG-*b*-PLAla hybrid system can be used as a chondrogenic differentiation platform of TMSCs. It was believed that the enhanced chondrogenic differentiation in the GO/mPEG-*b*-PLAla hybrid system was attributed to the increased cell adhesion on the 2D surfaces of GO and the interactions among GO, COL II and transforming growth factor β3 (TGF-β3).

A similar study was also reported by the same group. When arginyl-glycyl-aspartic acid (RGD)-coated hexagonal layered double hydroxides (LDHs) were incorporated into an mPEG-*b*-PLAla-*b*-poly(L-aspartate) (mPEG-*b*-PLAla-*b*-PLAsp) hydrogel [96], the chondrogenic biomarkers significantly increased compared with the pure hydrogel system. These studies suggested that 2D materials incorporated in the hydrogel can increase the rigidity of hydrogel, and provide additional adhesion sites for cells, which may facilitate cell proliferation and differentiation.

In addition, the modification of the thermogel with various functional groups has obvious influence on the differentiation of stem cells. Polystyrene (PS) microspheres modified with different functional groups were incorporated into an mPEG-*b*-PLAla thermogel [95]. It was reported that the thermogels incorporated with thiol-functionalized microspheres promoted adipogenesis and chondrogenesis of TMSCs *in vitro*, while those modified with phosphate or carboxylate facilitated chondrogenesis and osteogenesis. Additionally, the hydrogel embedded with ammonium-functioned microspheres promoted adipogenesis, while the neat microsphere-incorporated hydrogel facilitated osteogenesis. The study indicated that the microspheres modified by different functional groups can induce the preferential differentiation of stem cells into different cell types.

The effect of PLAla chain length on cell behavior was also investigated [93]. The study showed that the chain length variation may affect secondary structure arrangement, thus leading to variable microarchitecture and mechanical property. It was found that an increase in the PLAla chain length would promote cell cluster and support higher GAG and collagen deposition. It was thought that the chondrocyte phenotype maintaining and matrix production were related to secondary structure arrangement and fibrillar-like microarchitecture. Therefore, the study indicated that the polypeptide chain length can exhibit a significant influence on cell differentiation, through affecting the secondary conformation and self-assembly structure.

**Figure 6.**
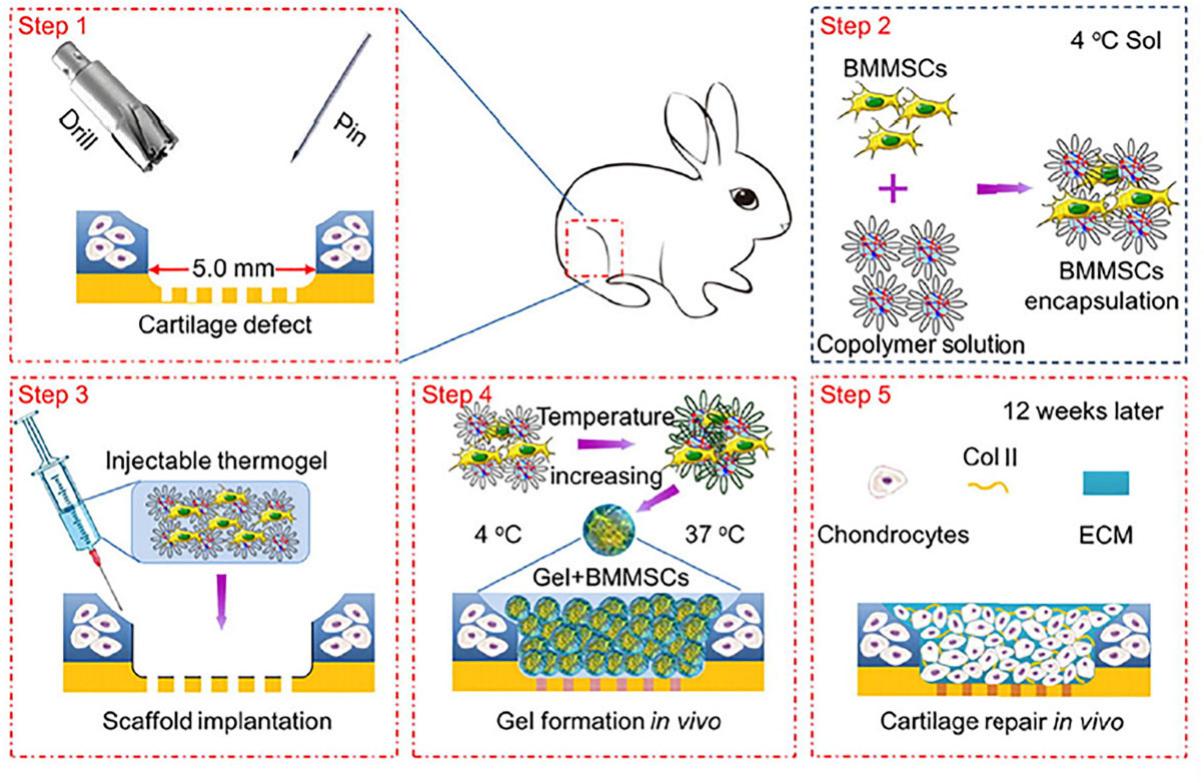
Schematic illustration for the thermo-induced hydrogel formation at cartilage defect by injection of the BMSC-encapsulated P(LAla-*co*-LPhe)-*b*-PEG-*b*-P(LAla-*co*-LPhe) hydrogel into the cartilage defect, and the subsequent cartilage repair *in vivo*. Reproduced with permission from Ref. [98].

In addition, mPEG-*b*-P(LAla-*co*-LPhe) and P(LAla-*co*-LPhe)-*b*-PEG-*b*-P(LAla-*co*-LPhe) thermosensitive hydrogels have also been investigated as 3D scaffolds for the differentiation of TMSCs and BMSCs [98, 106]. It was seen that chondrogenesis was also promoted in this type of hydrogels. The introduction of L-phenylalanine residues into polyalanine-based hydrogel resulted in larger pore size, lower CGC and higher mechanical strength. It was found that, after injection of BMSC-laden P(LAla-*co*-LPhe)-*b*-PEG-*b*-P(LAla-*co*-LPhe) hydrogels into osteochondral defect of New Zealand rabbits, enhanced levels of GAGs and Col II were generated compared with the control group at 12 weeks, suggesting the chondrogenic differentiation of the BMSCs (Fig. 6).

The PLAla-*b*-PLX-*b*-PLAla hydrogels have also been shown to promote chondrogenic differentiation. The injectable PLAla-*b*-PLX-*b*-PLAla copolymer solutions formed hydrogels *in situ* at 37°C, and were used as 3D scaffolds for chondrocyte culture [97]. It was found that the *β*-sheet structure increased as the L-alanine content increased, and the hydrogel with more *β*-sheet structure tended to develop more nanofibrous matrix than the hydrogel with higher content of random coiled structure. The fibrous structure was found to play a crucial role in cell proliferation since it can provide skeletal and mechanical environments in the ECM. The mechanical properties markedly influenced the proliferation and differentiation of the cells. It was found that the chondrocyte-encapsulated polypeptide hydrogel displayed higher levels of sGAG content, Col II expression and cell proliferation than commercially available Martrigel.

#### Bone tissue engineering

To construct scaffolds for osteogenesis from stem cells, osteoinductive and osteoconductive components have been introduced to the thermo-responsive polypeptide hydrogels. A kind of injectable meso-composite system consisting of mPEG-*b*-P(LAla-*co*-LPhe) thermogel and well-defined calcium phosphate mesocrystals (4–8 µm) was prepared by Jeong and co-workers [99]. After incubation of TMSCs for 21 days, higher mRNA and protein expression biomarkers of osteogenesis such as bone morphogenetic protein 2, ALP and osteocalcin were observed in the mesocrystal composite systems, compared with pure hydrogel systems or composite systems incorporating calcium phosphate nanoparticles (10–100 nm). In this study, the inorganic mesocrystals in the composites can provide hard surfaces to bind cells and proteins, whereas the mPEG-*b*-P(LAla-*co*-LPhe) hydrogel can serve as a soft matrix to hold the cells.

**Figure 7.**
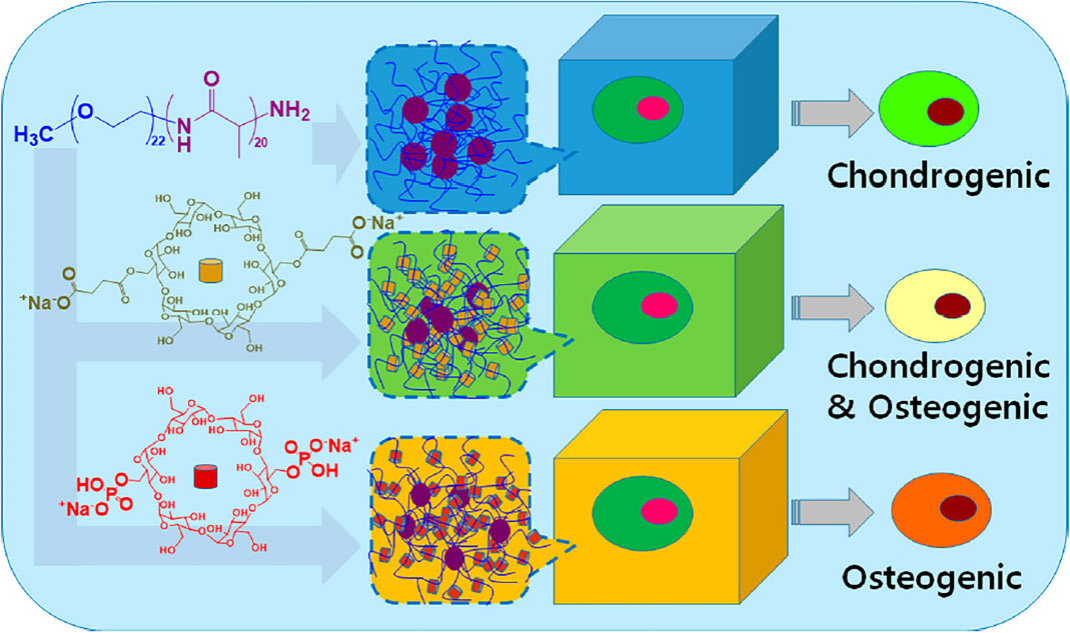
Schematic illustration for the differentiation of TMSCs in blank mPEG-*b*-PLAla hydrogel, mPEG-*b*-PLAla/*α*-CD carbonate inclusion complex hydrogel, or mPEG-*b*-PLAla/*α*-CD phosphate inclusion complex hydrogel. Reproduced with permission from Ref. [107].

In a subsequent study by Jeong and co-workers, it was found that the differentiation of TMSCs encapsulated within the thermosensitive polypeptide hydrogel could be regulated by introducing *α*-cyclodextrin (*α*-CD) derivatives containing carboxylate and phosphate groups, respectively [107]. It was demonstrated that inclusion complexes were formed after mixing mPEG-*b*-PLAla with *α*-CD derivatives, which also showed a thermo-induced sol–gel phase transition, with the storage moduli of the hydrogels ranging from 1.0 to 1.3 kPa. Compared with the chondrogenic differentiation of TMSCs in the blank polypeptide hydrogel, osteogenic differentiation of the stem cells was observed after incubation in the polypeptide/*α*-CD phosphate hydrogel for 21 days (Fig. 7).

In a separate study by Chu and co-workers, osteoinductive and osteoconductive Fe_3_O_4_ magnetic nanoparticles (MNP) and hydroxyapatite (HAP) nanoparticles were incorporated into mPEG-*b*-PLAla hydrogel [100]. HAP is known to promote osteoblast proliferation and enhance the ALP expression. When MC3T3-E1 pre-osteoblasts were encapsulated in the MNP-incorporated hydrogel and cultured for 21 days *in vitro* under a magnetic field, osteogenic differentiation was significantly enhanced. The strategy of combing magnetic field and magnetic-responsive hydrogel scaffolds can provide a promising platform to modulate the biological responses of cells.

In a recent study by Yu, Yu, Liu and co-workers, hypoxic extracellular vesicles (EVs) derived from BMSCs were encapsulated into an mPEG-*b*-PLAla hydrogel [108]. The hypoxic EVs-loaded polypeptide hydrogel showed sustained release of the EVs for 3 weeks *in vitro*. Following injection of the EV-laden hydrogel into rat cranial defects of 5 mm in size, accelerated bone regeneration was observed compared with the blank hydrogel and the hydrogel containing normal EVs. It was proposed that the biglycan-rich EVs generated through hypoxia preconditioning play an important role in modulating osteoblast differentiation and mineralization.

#### Neural tissue engineering

A series of physically crosslinked amphiphilic DCHs have been evaluated for CNS regeneration by Deming, Sofroniew and co-workers, since the hydrogels showed comparable storage moduli to native brain tissues [32]. For instance, DCHs composed of L-lysine and L-leucine residues (PLLys-*b*-PLLeu) was synthesized and the toxicity against CNS was tested, because L-lysine-based materials are usually applied as substrates for culture of nerve cells. It was found that the diblock copolypeptide exhibited no detectable or little toxicity in the CNS and integrated with the brain after injection into the brain of C57BL/6 mice.

Furthermore, the DCH was used for sustained release of bioactive proteins, such as nerve growth factor (NGF) [109]. To evaluate the *in vivo* delivery of NGF, mouse forebrain cholinergic neurons size was measured and the storage modulus of DCH was tuned to just below the modulus of brain tissue. Compared with NGF solution in buffer, NGF-loaded copolypeptide hydrogel provided a much longer release profile of NGF for at least 4 weeks. It was seen that NGF-loaded hydrogel caused hypertrophy of forebrain cholinergic neurons 5 mm away from the injection site. Thus, the DCH provided an effective approach to deliver bioactive proteins to CNS, with a sustained release profile and thus predicted efficacy on local cells.

In addition, small hydrophobic molecules such as temozolomide and Tamoxifen were incorporated into DCH for *in vitro* and *in vivo* drug delivery [110]. The study demonstrated that the loading capacity and release rate can be tuned by simply changing DCH formulations. Furthermore, Tamoxifen released from DCH injected into CNS can efficiently activate reporter gene expression in transgenic mice. Therefore, DCH showed potential as carrier for sustained delivery of small hydrophobic drugs in CNS.

**Figure 8.**
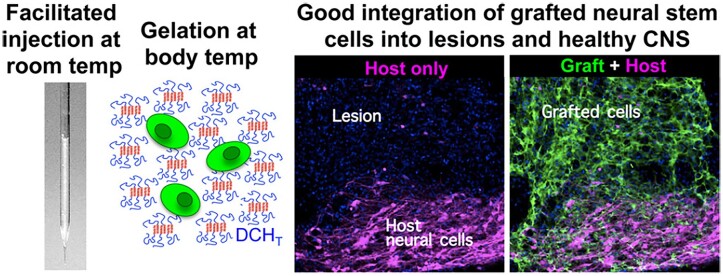
Injectable and thermoresponsive DCH_T_ as vehicles for transplantation of NSCs into CNS. Reproduced with permission from Ref. [101].

In addition to ionic DCH containing a poly(glutamic acid) or polylysine block as the hydrophilic building block, nonionic DCH has also been investigated for drug delivery in CNS. A nonionic and thermo-responsive DCH based on a nonionic, hydrophilic polypeptide block with OEG side groups ((*rac*-E^P2^)_m_-(L_x_/$E_{1-x}^{P2}$)_n_, denoted as DCH_T_) was prepared and used as a vehicle for sustained drug delivery in CNS [101]. DCH_T_ exhibited excellent cytocompatibility while maintaining injectability, tunable rigidity, porosity and the capacity of delivering both hydrophobic and hydrophilic molecules in CNS. It was found that the neural stem cells (NSCs) delivery by DCH_T_ integrated well into the lesions and healthy CNS (Fig. 8).

Nonionic poly(L-methionine sulfoxide) were incorporated into the DCHs (DCH_MO_) as the hydrophilic block, due to its superior anti-fouling property, which was studied as a scaffold for neural stem/progenitor cell (NSPC) transplantation [111]. It was found that non-fouling DCH_MO_ exhibited a better ability to preserve the stemness and multipotency of NSPC than a cationic copolypeptide hydrogel. The *in vivo* transplantation study by injection into caudate putamen nucleus of C57BL/6 mice showed that NSPC-encapsulated in DCH_MO_ remained an immature astroglial phenotype and integrated well with host neural tissue, and supported the growth of host-derived axons. Overall, the DCH has shown good biocompatibility as well as the ability to deliver small molecules, proteins and NSPC in CNS.

To further adjust the release behavior of bioactive agents, hierarchical drug delivery systems have been designed. A microsphere-incorporated thermo-responsive hydrogel was developed by Jeong and co-workers for controlled delivery of brain-derived neurotropic growth factor (BDNF) and NGF [102]. BDNF and NGF were loaded separately into alginate microspheres, followed by incorporating the microspheres in an *in situ* formed PEG-*b*-PLAla hydrogel with increasing temperature. It was shown that these two growth factors were released sustainedly over 12–18 days, and the TMSCs incorporated in the hydrogel exhibited a multipolar elongation during the culture within the 3D environment. It was observed that the dual barriers of microsphere and hydrogel efficiently decreased the initial burst release of proteins.

#### Skin tissue engineering

As a platform for 3D cell culture, the thermo-responsive mPEG-*b*-PLAla hydrogel encapsulating fibroblasts was investigated for skin wound healing [103]. The cell-laden hydrogel can be formed *in situ* by thermo-induced sol–gel transition of the mPEG-*b*-PLAla solution in PBS. When cultured *in vitro*, the cell-encapsulated hydrogel showed significantly higher expressions of collagen types I and III, compared with commercially available Matrigel. After applying to a full-thickness round wound of 10 mm in diameter of rats, the fibroblast-encapsulated hydrogel was found to promote wound closure, achieve effective epithelialization and facilitate generation of skin appendages. The results demonstrated that the thermo-responsive polypeptide hydrogel has potential as a scaffold for cell delivery to skin wound sites.

#### Liver tissue engineering

Due to the temperature-dependent sol–gel phase transition, various small molecules, proteins and cells can be simultaneously encapsulated in the thermo-responsive hydrogels, through mixing the agents and cells with the polymer aqueous solution at lower temperatures, followed by facilely increasing the temperature. In a study by Jeong and co-workers, tauroursodeoxycholic acid (TUDCA), fibroblast growth factor 4 (FGF4), hepatocyte growth factor (HGF) and TMSCs were simultaneously incorporated into a thermo-responsive mPEG-*b*-PLAla hydrogel to induce the hepatogenic differentiation of TMSCs [104]. The small molecule and proteins encapsulated within the hydrogel exhibited sustained release profiles over 21 days *in vitro*. During the culture of 21 days *in vitro*, the TMSCs showed a predominantly spherical morphology with some elongated shape. The mRNA expressions and protein production related to hepatogenic differentiation biomarkers were significantly higher in the cell-encapsulated thermogel than those in a commercially available hyaluronic acid gel. In addition, the hepatocytes differentiated from the TMSCs in the hydrogel displayed hepatic biofunctions comparable to HepG2 cells, such as the uptakes of low-density indocyanine green (76%) and lipoproteins (52%), as well as the production of urea (52%) and albumin (40%).

**Figure 9.**
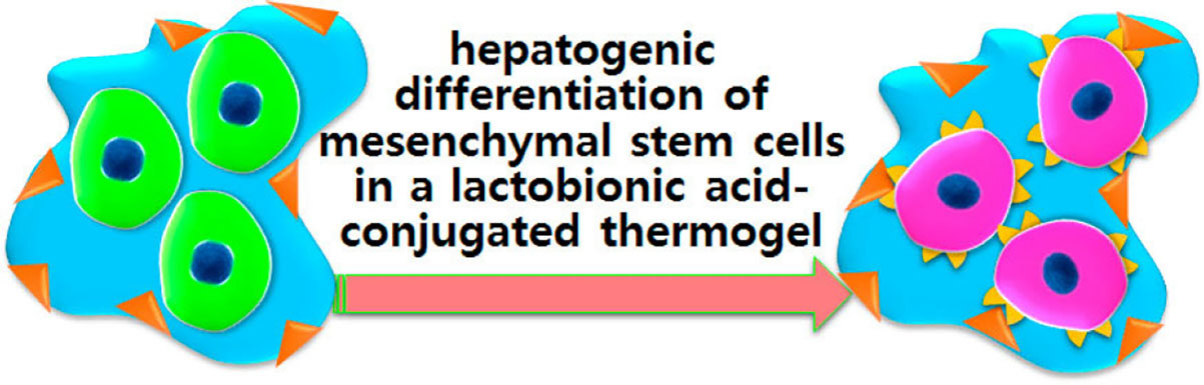
Design of a bioactive 3D scaffold. Reproduced with permission from Ref. [105].

Except for introduction of growth factors, receptor substrates of stem cells were also reported to be positive for inducing stem cell differentiation. For example, lactobionic acid (LB) can increase albumin secretion and urea production of hepatocyte, due to its high binding affinity to asialoglycoprotein receptors (ASGR) on the surface of hepatocytes [105]. Accordingly, an LB-conjucted PLX-*b*-PLAla (LB-PLX-*b*-PLAla) block copolymer was prepared, and the resultant thermo-responsive hydrogel was studied as a scaffold for hepatogenic differentiation of TMSCs (Fig. 9). When cultured *in vitro* for 23 days, TMSCs in the LB-PLX-*b*-PLAla hydrogel retained a predominantly spherical morphology, in contrast to obvious morphological change of those in Matrigel. Additionally, it was found that TMSCs encapsulated within the LB-conjucted polypeptide hydrogel showed enhanced expressions of hepatogenic biomarkers such as albumin, hepatocyte nuclear factor 4α (HNF4α), and ASGR, compared with those in the blank PLX-*b*-PLAla hydrogel and Matrigel. In addition, the increased generation of albumin and urea of the differentiated TMSCs was further confirmed. Therefore, the modification of receptor substrates into hydrogel scaffolds can also be an effective approach for modulation of stem cell proliferation and differentiation.

#### Islet tissue engineering

Islet transplantation has attracted wide attention for its potential in treating type 1 diabetes. The synthetic polymers for islet encapsulation such as poly(lactic acid) (PLA), poly(lactic-co-glycolic acid) (PLGA) and poly(vinyl alcohol) enquire either pretreated scaffolds or surgical procedures for transplantation. Since injectable hydrogels can be easily injected into transplantation sites and closely mimic native ECM, they become a kind of particularly attractive material for islet transplantation.

In a recent study by Chu and co-workers [112], MIN6 β cells derived from insulinoma were encapsulated in a PEG-*b*-PLAla hydrogel, to investigate the feasibility of using this type of thermo-responsive hydrogel as a scaffold for islet transplantation. It was seen that the MIN6 β cells maintained viability and capacity of insulin secretion when the cell-encapsulated hydrogel was cultured *in vitro*. After subcutaneous injection into nude mice, the cells within the hydrogels could survive and recreate insulin at 14 days post-implantation.

### Polypeptide hydrogels for localized drug delivery

Anticancer drug delivery systems have received extensively investigation in the past several decades, especially those designed for the delivery of chemotherapeutic drugs, proteins and immunotherapeutic agents. However, it is known that the systemic administration of chemotherapeutic and immunotherapeutic drugs may lead to insufficient drug concentration at targeted sites and serious systemic side effects. Additionally, the maintaining of bioactivity of many bioactive agents such as proteins has been challenging.

Recently, chemotherapy and immunotherapy based on the hydrogel systems have been explored for cancer treatment. For instance, various chemotherapeutic drugs including 5-fluorouracil (5-FU), cisplatin (CDDP), doxorubicin (DOX) and paclitaxel (PTX) have been loaded in hydrogels, and the drugs can be released in a sustained manner at the injection sites following injection to the targeted sites. Additionally, immunotherapeutic agents such as cytokines, immune checkpoint blockading (ICB) agents and immune cells have also been incorporated into hydrogel systems to construct topic delivery systems. The hydrogel-based delivery systems are able to improve the bioavailability of the therapeutic agents at pathological sites or targeted sites, and markedly reduce the systemic side effects of the agents [113, 114].

Due to their good biocompatibility, biodegradability and the temperature-dependent sol–gel transitions, some thermo-responsive polypeptide hydrogels have been developed for localized, sustained delivery of various anticancer agents, including chemotherapeutic drugs, tumor antigens, cytokines, adjuvants and ICB antibodies. Several representative systems as the local drug delivery depots are listed in Table 3.

**Table 3.** Typical applications of thermosensitive polypeptide hydrogels in drug delivery

Sample codes	Drugs	*In vivo* test	Potential application	Ref.
PELG-*b*-PEG-*b*-PELG	PTX	BALB/c nude mice xenoimplanted with HepG2 cells	Chemotherapy	[115]
	DOX, IL-2, IFN-γ	B16F10 melanoma xenograft in a mice model	Chemoimmunotherapy	[116]
mPEG-*b*-PLVal	TCLs, poly(I: C)	Female C57BL/6 mice inoculated with B16 melanoma	Immunotherapy	[26]
mPEG-*b*-PLAla	TCL, GM-CSF, anti-CTLA-4, anti-PD-1	C57BL/6 mice bearing B16F10 melanoma or 4 T-1 tumor	Immunotherapy	[117]
P(LMet-*co*-D-1MT)-*b*-PEG-*b*-P(LMet-*co*-D-1MT)	anti-PD-L1	C57BL/6 mice bearing B16F10 melanoma	Immunotherapy	[86]
mPEG-*b*-PELG	CDDP, IL-15	C57BL/6 mice bearing B16F0-RFP melanoma	Chemoimmunotherapy	[118]
	CDDP, CA4P	BALB/c mice bearing C26 xenograft models	Chemotherapy	[119]
	DOX, D-1MT, anti-PD-1	C57BL/6 mice bearing B16F10 melanoma	Chemoimmunotherapy	[120]
	DOX, anti-CTLA-4, anti-PD-1	C57BL/6 mice bearing B16F10 melanoma	Chemoimmunotherapy	[121]
mPEG-*b*-P(LAla-*co*-LPhe)	rhGH	Rats	Growth hormone deficiency	[122]

#### Hydrogels for delivery of chemotherapeutic drugs

Chen and co-workers synthesized a poly(γ-ethyl-L-glutamate)-*b*-PEG-*b*-poly(γ-ethyl-L-glutamate) (PELG-*b*-PEG-*b*-PELG) triblock copolymer through the ROP of γ-ethyl-L-glutamate NCA (ELG NCA) using amino-terminated PEG as the macroinitiator [115]. The 3–5 wt% PELG-*b*-PEG-*b*-PELG aqueous solutions displayed heat-induced sol–gel phase transitions with the gelation temperatures of 15–33°C. A hydrophobic chemotherapeutic drug, PTX, was loaded into the amphiphilic block copolymer hydrogel as a drug depot for local sustained delivery. The PTX-loaded hydrogels showed sustained drug release profiles for over 3 weeks *in vitro*. In the subcutaneous layer of rats, the polypeptide hydrogel lasted for over 2 weeks. Following peritumoral injection of the PTX-loaded hydrogel into BALB/c nude mice bearing HepG2 xenograft, the treatment showed obvious suppression on the tumor growth for up to 21 days, and the tumor inhibition efficacy was found to be higher than the local treatment with Taxol solution.

It has been established that relatively hydrophilic chemotherapeutic drugs, such as DOX and CDDP, show rapid diffusion rate in aqueous solutions, leading to initial burst release from hydrogel depots [123]. To achieve a prolonged CDDP release, an injectable thermo-responsive hydrogel based on methoxy-poly(ethylene glycol)-*b*-(poly(*γ*-ethyl-L-glutamate-*co*-L-glutamic acid) (mPEG-*b*-P(ELG-*co*-LG)) was prepared for the formation of complexation between CDDP and the carboxyl groups of polypeptide [124]. Compared with the CDDP-loaded mPEG-*b*-PELG hydrogel without complexation, the burst release of CDDP was obviously inhibited *in vitro*. After peritumoral injection into C26 tumor-bearing BALB/c mice, the CDDP-complexed mPEG-*b*-P(ELG-*co*-LG) hydrogel exhibited significantly enhanced tumor inhibition efficacy *in vivo*, compared with free CDDP solution or CDDP-loaded mPEG-*b*-PELG hydrogel. The results suggested that the prolonged CDDP release at tumor site could effectively prolong the tumor inhibition efficacy.

**Figure 10.**
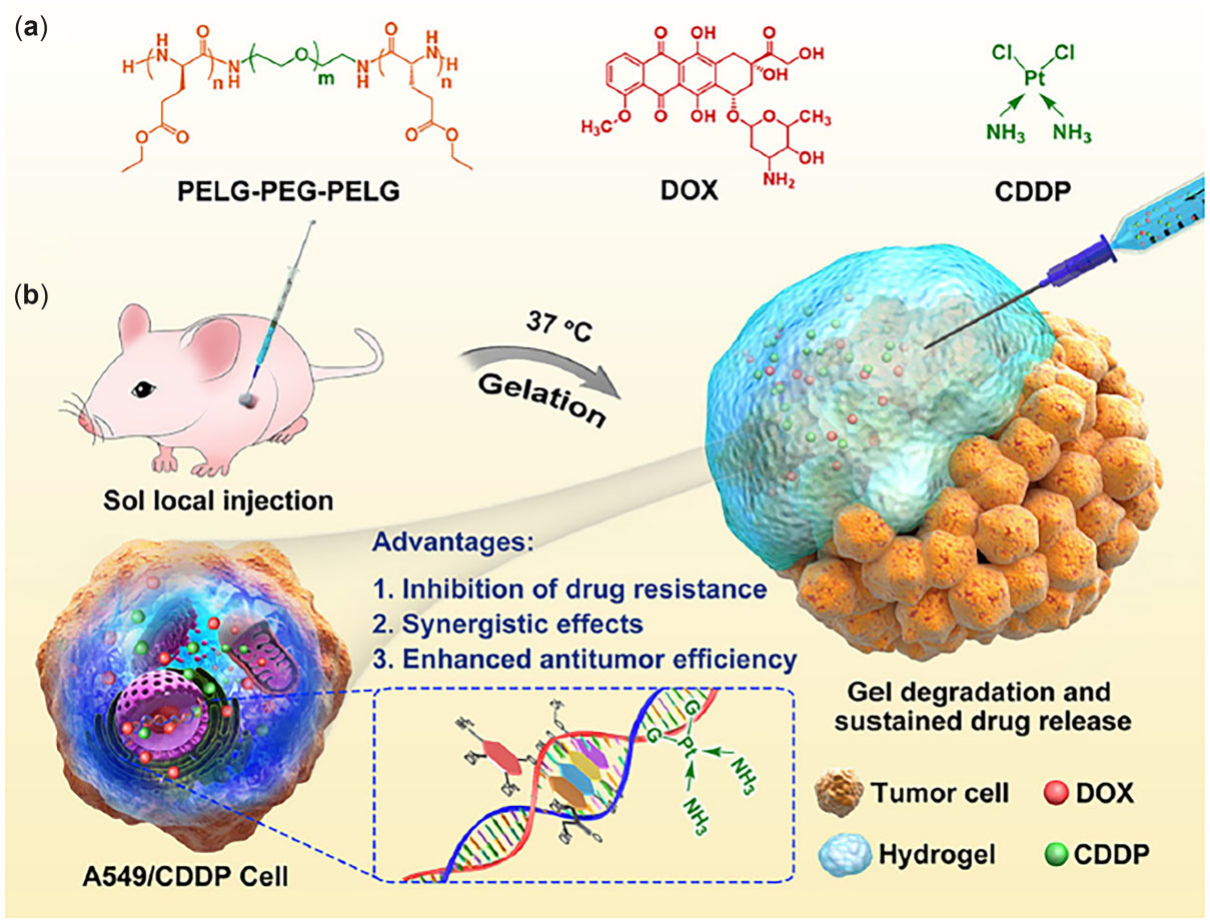
DOX/CDDP co-Loaded PELG-*b*-PEG-*b*-PELG hydrogel for local tumor combination chemotherapy. (**a**) Chemical structures. (**b**) Local combination treatment of the nude mouse MDR tumor model. Reproduced with permission from Ref. [126].

Injectable hydrogels have also been investigated as platforms for co-delivery of dual or multiple agents for tumor combination therapy, due to their enhanced antitumor efficacy, increased inhibition of drug resistance, reduced drug dosages and relieved toxic side effects [125]. He, Chen and co-workers encapsulated DOX and CDDP in the PELG-*b*-PEG-*b*-PELG thermosensitive hydrogel as a system for local tumor combination chemotherapy [126]. It was found that the simultaneous release of DOX and CDDP from the hydrogel exhibited synergistic effects in the inhibition of A549 and drug-resistant A549/CDDP tumor cells *in vitro*. Moreover, the DOX/CDDP co-loaded hydrogels displayed significantly higher antitumor efficacy than the DOX/CDDP solution or hydrogels loaded with a single drug, following peritumoral injection into A549 xenograft-bearing nude mice (Fig. 10).

In addition to the systems for co-delivery of dual chemotherapeutic drugs, the combination of chemotherapeutic drug and the agents targeting the tumor microenvironment (TME) has also received progressive attention. For instance, an injectable microsphere-incorporated hydrogel system was fabricated by encapsulating combretastatin A-4 (CA4) and docetaxel (DTX)-loaded PLGA microspheres into the P(LAla-*co*-LPhe)-*b*-PEG-*b*-P(LAla-*co*-LPhe) hydrogel for the sequential delivery of CA4 and DTX [127]. It was designed that, after injection into tumor site, the vascular disrupting agent, CA4, was first released from the hydrogel to destroy the blood vessels and block the supply of nutrients for tumor cells, while DTX could be released from the microsphere-embedded hydrogel in a more sustained manner to inhibit tumor cell proliferation, resulting the combination inhibition of osteosarcoma. During the peritumoral treatment of K7 osteosarcoma-bearing BALB/c mice, the CA4/PTX co-loaded hydrogel exhibited significantly higher antitumor efficacy compared with free CA4/PTX mixed solution and the hydrogel loaded with CA4 or DTX-encapsulating microspheres. In a subsequent study by He, Gu and co-workers, a thermosensitive mPEG-*b*-PELG hydrogel co-loaded with CDDP and CA4 disodium phosphate (CA4P) was developed for tumor combination treatment. The enhanced antitumor efficacy of the CDDP/CA4P co-loaded hydrogel was demonstrated in treatment of mice bearing C26 colon tumors with various tumor volumes (∼110 or 370 mm^3^), after peritumoral injection into the tumor-bearing BALB/c mice [119].

#### Hydrogels for co-delivery of chemotherapeutic drugs and cytokines

The past decade has witnessed the rapid progress of cancer immunotherapy. In clinical cancer immunotherapy, cytokines or recently developed immune checkpoint blockade (ICB) agents have been widely used. Cytokines are proteins that are involved in the growth, differentiation and activation of immune cells and each aspect of immunity and inflammation [128]. Additionally, some cytokines can directly induce tumor apoptosis *via* different mechanism. However, it has been reported that the systemic administration of cytokines or ICB antibodies may lead to severe systemic toxic side effects [113]. Thus, localized delivery systems based on injectable hydrogels have been developed for sustained delivery of the immunotherapy agents. Additionally, other antitumor agents, such as chemotherapeutic drugs, can also be incorporated into these systems for enhanced combination therapy.

In recent years, injectable thermosensitive polypeptide hydrogels have been investigated as depots for co-delivery chemotherapy drugs and different cytokines for cancer combination therapy. He, Chen and co-workers developed an mPEG-*b*-PELG hydrogel co-loaded with interleukin-15 (IL-15) and CDDP for tumor immuno-chemotherapy [118]. IL-15 is a cytokine that can promote the proliferation and differentiation of T cells and natural killer (NK) cells. The synergistic anticancer efficacy of CDDP and IL-15 was obtained by a single peritumoral injection of the IL-15/CDDP co-loaded hydrogel into B16F0-RFP melanoma-bearing C57BL/6 mice. It was found that IL-15/CDDP-induced activation and amplification of CD8^+^ T cell and NK cell led to reduced immunosuppression and enhanced antitumor immune response. In addition, CDDP-mediated S arrest significantly augmented the antitumor efficacy.

In another study by the same group, a PELG-*b*-PEG-*b*-PELG hydrogel co-loaded with IL-2, interferon-γ (IFN-γ) and DOX was also developed for chemoimmunotherapy of melanoma [116]. IL-2 is a cytokine that can mediate the proliferation of lymphocytes, including T cells and NK cells, while IFN-γ is able to stimulate antigen presentation, cytokine production and the effector functions of monocytes. Additionally, IL-2 and IFN-γ can directly induce the apoptosis of tumor cells through various pathways [129]. After peritumoral injection of the drug-loaded hydrogel into melanoma-bearing mice, an enhanced tumor inhibition efficacy was obtained compared with the hydrogel loaded with DOX or cytokines only. The strengthened anticancer efficacy was likely related to the increased proliferation of CD8^+^ and CD4^+^ T lymphocytes, as well as chemotherapy-induced tumor cell apoptosis.

#### Hydrogels for co-delivery of ICB antibodies and combined agents

Remarkable clinical outcomes have been achieved in cancer immunotherapy of several types of cancers based on ICB antibodies, especially those blockading the programmed cell death protein 1/programmed death-ligand 1 (PD-1/PD-L1) or cytotoxic T lymphocyte antigen 4 (CTLA-4) pathway. However, the clinical antitumor efficacies of ICB-based immunotherapy for a wide range of cancers are still limited by major drawbacks, including variable objective response rates (ORRs) and serious immune-related adverse events after systemic administration [113]. To improve the ORRs of ICB-based immunotherapy, various strategies such as the modulation of immunosuppressive TME and enhancement of antigen presentation have been investigated. Additionally, injectable hydrogels have been utilized as depots for the sustained, localized release of ICB antibodies, to relieve their systemic side effects.

**Figure 11.**
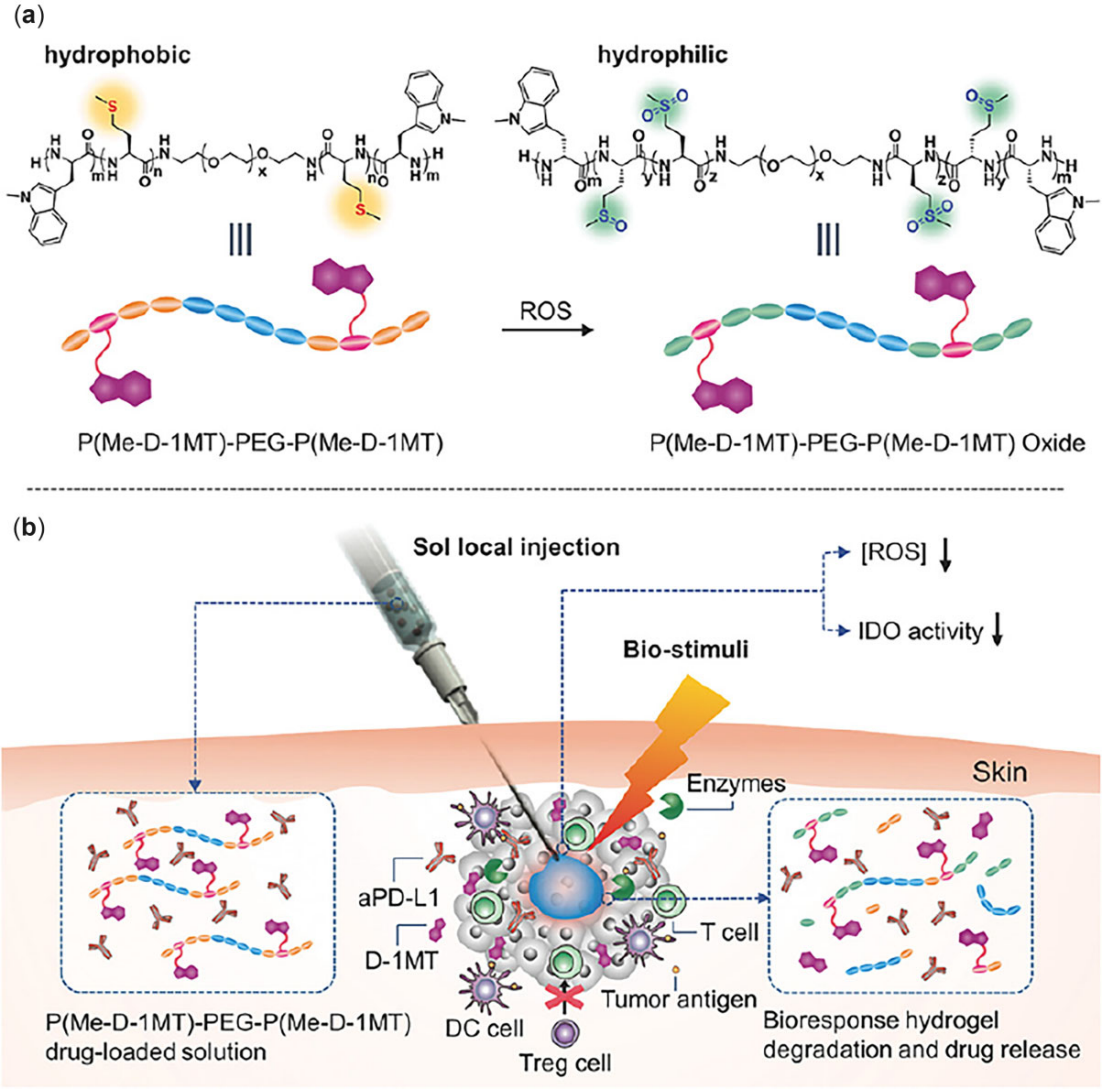
Synergistic immunotherapy of ROS-responsive P(LMet-*co*-D-1MT)-*b*-PEG-*b*-P(LMet-*co*-D-1MT). (**a**) Hydrophobicity transition and chemical structure and (**b**) localized drug delivery for synergistic immunotherapy. Reproduced with permission from Ref. [86].

It has been established that the high levels of ROS and indoleamine-2,3-dioxygenase (IDO) in the TME can hamper the survival and activation of T cells. To modulate the immunosuppressive TME, a bio-responsive P(LMet-*co*-D-1MT)-*b*-PEG-*b*-P(LMet-*co*-D-1MT) hydrogel was fabricated by co-polymerization of ROS-responsive LMet and a IDO inhibitor, D-1MT, into the polypeptide backbone [86]. The hydrogel loaded with anti-PD-L1 was investigated as a depot for tumor immunotherapy. Following intratumoral injection of the anti-PD-L1-loaded hydrogel into melanoma-bearing C57BL/6 mice, the levels of ROS and IDO in the TME can be reduced due to the anti-oxidation effect of LMet residues and IDO inhibiting effect of D-1MT that was gradually released by the degradation of the polypeptide segment (Fig. 11). The anti-PD-L1-loaded hydrogel showed higher antitumor efficacy compared with the free D-1MT/anti-PD-L1 mixed solution, which may be attributed to the prolonged drug/antibody release profiles of the hydrogel depot. The enhanced infiltration of CD8^+^ T cells in the tumors of the anti-PD-L1-loaded hydrogel group was demonstrated, suggesting an increased antitumor immune response.

In a similar study by Luan and co-workers, an immunomodulatory mPEG-*b*-PLNVal hydrogel was fabricated by the ROP of L-norvaline NCA using mPEG-NH_2_ as the macroinitiator [88]. It was designed that the sustained release of L-norvaline, an arginase 1 inhibitor, during the enzymatic degradation of the hydrogel could inhibit arginase 1 pathway and regulate the immunosuppressive TME. Following intratumoral injection into B16F10-bearing mice, the DOX-loaded mPEG-*b*-PLNVal hydrogel showed inhibition effect on the arginase 1 in tumor and strengthened the antitumor immune response, which was elicited by DOX-induced immunogenic cell death (ICD) of tumor cells.

In a subsequent study by He and co-workers, DOX, D-1MT and anti-PD-1 were co-loaded into an mPEG-*b*-PELG hydrogel for antitumor chemoimmunotherapy [120]. It was found that the sustained release of DOX caused the ICD of B16F10 cells *in vitro*, leading to the release of tumor-associated antigens (TAAs) and damage-associated molecular patterns (DAMPs), such as exposure of calreticulin to cell membranes, ATP secretion and high mobility group box 1 (HMGB1) release. The lysates of tumor cells could cause the activation of dendritic cells (DCs), which may promote the antigen presentation to T lymphocytes. After intratumoral injection of the drug-loaded hydrogel into melanoma-bearing C57BL/6 mice, the PD-1/PD-L1 pathway for immune escape of tumor cells could be blockade by the sustained release of anti-PD-1. Additionally, the sustained release of D-1MT could promote the activity of T cells. The DOX/D-1MT/anti-PD-1 co-loaded hydrogel exhibited higher antitumor efficacy, extended the animal survival time and elicited stronger antitumor immune response, compared with the DOX/D-1MT/anti-PD-1 solution and the hydrogel loaded with DOX only.

He and co-workers also encapsulated DOX, dual ICB antibodies, anti-CTLA-4 and anti-PD-1, into the mPEG-*b*-PELG hydrogel as a system for tumor immunotherapy and prevention of post-surgical tumor reoccurrence [121]. Through peritumoral injection of the DOX/anti-CTLA-4/anti-PD-1 co-loaded hydrogel into melanoma-bearing C57BL/6 mice, the ICD of tumor cells by DOX release caused the release of DAMPs and TAAs, which can promote the activation of DCs. Additionally, the sustained release of anti-CTLA-4 could block the CTLA-4/B7 pathway and thus promote the antigen presentation of activated DCs to T cells. Further, during the tumor killing stage by cytotoxic T lymphocytes (CTLs), the PD-1/PD-L1 pathway for immune escape of tumor cells was inhibited by the local release of anti-PD-1. In the *in vivo* antitumor study, the DOX/anti-CTLA-4/anti-PD-1 co-loaded hydrogel caused higher tumor inhibition efficiency, prolonged mice survival time and increased infiltration of CD8^+^ T cells in tumors, compared with the hydrogel loaded with DOX or antibodies only. Moreover, in the tumor resection mice model, the multi-agent-loaded hydrogel showed significantly higher efficacy in inhibiting tumor reoccurrence, compared with the DOX/anti-CTLA-4/anti-PD-1 mixed solution.

#### Hydrogels for delivery of tumor antigens

It has been reported that antigen-encapsulated nanoparticles and 3D scaffold can modulate host DC populations and enhance the anti-tumor efficiency through controlled release of biochemical molecules, such as antigens, adjuvant and cytokines [117]. Wang and co-workers developed a vaccine strategy by using the mPEG-*b*-PLVal hydrogel as the scaffold to encapsulate tumor cell lysates (TCLs) and a toll-like receptor 3 (TLR3) agonist, polyinosinic:polycytidylic acid (poly(I:C)) [26]. TLR agonists are effective vaccine adjuvants that can stimulate the activation of DCs. The hydrogel realized the sustained release of TCLs and poly(I:C) for more than 1 week, and promote the antigen drainage to lymph nodes following subcutaneous injection into BALB/c mice. After subcutaneous injection into the region adjacent to the B16 melanoma (two times), the TCLs/poly(I:C) co-loaded hydrogel showed significantly higher tumor inhibition efficiency than the TCLs/poly(I:C) solution or TCLs-loaded hydrogel. The enhanced infiltration of CD8^+^ T lymphocytes into the tumors treated with the TCLs/poly(I:C) co-loaded hydrogel was demonstrated, indicating the strengthened antitumor immunity.

In another study by the same group, a vaccine strategy was developed by encapsulating TCLs, granulocyte-macrophage colony-stimulating factor (GM-CSF), anti-PD-1 and anti-CTLA-4 into an mPEG-*b*-PLAla hydrogel [130]. GM-CSF was used as an adjuvant to help DCs recruitment and activation. It was found that the *in vivo* tumor inhibition efficacy and antitumor immune response can be further augmented by the incorporation of anti-PD-1 and/or anti-CTLA-4.

#### Polypeptide hydrogels for sustained release of growth hormone

An mPEG-*b*-P(LAla-*co*-LPhe) diblock copolymer hydrogel was used for controlled release of recombinant human growth hormone (rhGH), which can be used to treat children or adults with growth hormone deficiency [122]. The sustained release of the rhGH for up to 6 days with a slight initial burst release was realized. A pH- and temperature-responsive hydrogel of poly[(2-(dibutylamino)ethyl-L-glutamate)-*co*-(γ-benzyl-L-glutamate)]-*b*-PEG-*b*-poly[(2-(dibutylamino)ethyl-L-glutamate)-*co*-(γ-benzyl-L-glutamate)] [131] was also prepared for the sustained delivery of hGH. The hGH-loaded polypeptide hydrogel showed reduced initial burst, with a sustained release profile for 1 week after subcutaneous injection into Sprague Dawley (SD) rats. These studies suggest that the polypeptide hydrogels can be a promising platform for sustained delivery of proteins.

### Polypeptide hydrogels for anti-bacteria

The bacterial infection from surgical procedures and trauma may result in life-threatening sepsis. The commonly used antibiotics, antiseptics, wound dressings and bandages cannot meet the demand for prevention of wound infection. Thus, hydrogels with antimicrobial properties can be promising candidates for preventing wound infection. Deming et al. prepared a kind of hydrogel composed of poly(L-lysine·hydrochloride)_100_-*b*-poly(L-leucine)_40_ (PLLys_100_-*b*-PLLeu_40_) diblock copolypeptides [132]. The direct antimicrobial activity and microbial barrier properties of the PLLys_100_-*b*-PLLeu_40_ hydrogel made it broadly effective in inhibiting common wound pathogens such as Gram-positive and Gram-negative bacteria. In the porcine open wound model, a single treatment of 2 wt% DCH for 15 min before inoculation of *Staphylococcus epidermidis* led to 99.99% decline in colony forming unit (CFU) of the bacteria. Additionally, in an SD rat closed wound model, the pretreatment of a polypropylene mesh implant and the wound pocket with the diblock copolypeptide aqueous solution before inoculation of methicillin resistant *Staphylococcus aureus* (MRSA) resulted in 4–5 log decline in CFU of MRSA. These results indicated good antibacterial efficacies of the diblock copolypeptide against different types of bacteria *in vitro* and *in vivo*.

## Conclusions and perspectives

In recent years, several types of thermo-induced physically crosslinked polypeptide hydrogels have been designed and fabricated, including amphiphilic diblock copolypeptides and PEG/polypeptide block copolymers. Through the molecular design, the temperature regions of sol–gel phase transitions for these polypeptide block copolymers could be controlled in the range of 10–40°C, which were near the physiological temperature. The thermo-induced gelation mechanisms involve variation of secondary conformation, increased inter-molecular interactions, reduced hydration of PEG block, as well as enhanced chain entanglement and micellar aggregation. The polypeptide hydrogels showed relatively lower CGCs of 1–10 wt%, which may be due to their unique inter-molecular hydrogen-bonding mediated by secondary conformation. Owing to their reversibly physically crosslinked networks, the hydrogels showed moderate storage moduli ranging from 0.1 to 1 kPa. The polypeptides exhibited good biocompatibility, and the biodegradation mediated by erosion and enzymatic cleavage of polypeptide segments lasted for 1–5 weeks.

Through encapsulation of various bioactive agents, cells or drugs, the polypeptide hydrogels may be utilized as different platforms for biomedical applications. For instance, polypeptide hydrogels have been studied as scaffolds for *in situ* modulation of the differentiation of stem cells. On the other hand, a wide range of antitumor agents, including chemotherapeutic drugs, cytokines, ICB antibodies, adjuvants and TAAs, have been incorporated into the polypeptide hydrogels for local tumor treatment and combination therapy. These systems have several advantages including high concentrations of antitumor agents at tumor sites for a prolonged period and markedly reduced systemic side effects. Additionally, attributed to the functional side groups of the amino acid residues, polypeptide hydrogels with additional biofunctions, such as antibacterial activity, have also been developed through the introduction of cationic side groups and hydrophobic groups in the polypeptide segments. Overall, considerable polypeptide hydrogels have shown strong potential for different biomedical applications.

However, there are still some challenges for the practical applications of polypeptide hydrogels. As a scaffold for cell culture and tissue engineering, the physically crosslinked polypeptide block copolymer hydrogels showed good biocompatibility and dynamic crosslinking property. However, the mechanical strength of these hydrogels may need to be further improved to maintain the stability of the scaffold during the long-term cell proliferation, differentiation and migration.

Second, polypeptide hydrogels with stimuli-responsiveness still have room to be improved for precise applications *in vivo*. Since there are a series of chemical gradients in tumor TME, such as gas gradient (O_2_), systemic or local growth factor gradient (hormones or cytokines), metabolite gradient (glucose or lactate) and pH gradient, which is relatively complicated and may be much more ‘smarter’ than well-designed stimuli-sensitive hydrogels. Thus the expected *in vivo* antitumor effects may not be achieved in many cases. Moreover, the reversible stimuli-responsiveness may have advantages in some applications, such as the systems that exhibit reversible responsiveness to thermal, light or specific biomolecules. The reversible responsiveness can lead to the sol–gel–sol phase transition of hydrogels, and make them more intelligent and flexible to control the cargo loading and release in an on–off switch manner.

Third, most of the current polypeptide block copolymer hydrogels are lack of specific biofunctions. Biofunctionalized polypeptide hydrogels may be of interest for not only 3D cell culture and drug delivery. Appropriate interactions between the hydrogel scaffolds and encapsulated cells can have advantages for maintaining the bioactivity and functions of cells, and for better control of cell behaviors within the hydrogels. Additionally, as drug carriers for tumor treatment, bioactive functions of polypeptide hydrogels, such as the capacity of modulating the TME, ability to recruit immune cells and even direct tumor-killing activity, can markedly improve the antitumor outcome of the payloads.

Finally, attributed to their good biocompatibility of polypeptide block copolymers and their degradation products, several PEG-*b*-polypeptide diblock copolymers including mPEG-*b*-PLGlu and mPEG-*b*-(α,β-aspartic acid) have been evaluated as drug carriers for tumor therapy in Phases I–III clinical trials [133, 134]. For instance, a cisplatin-loaded mPEG-*b*-PLGlu micelle (NC-6004) has been tested in phase I/II clinical trials in USA and Europe, as well as Phase III clinical trials in Eastern Asia for the treatment of different types of cancers. Nevertheless, to achieve the clinical translation of polypeptide block copolymer hydrogels, comprehensive preclinical studies and clinical trials are needed.

## References

[1] He C , Zhuang X, Tang Z, Tian H, Chen X. Stimuli-sensitive synthetic polypeptide-based materials for drug and gene delivery. Adv Healthc Mater 2012;1:48–78. 23184687 10.1002/adhm.201100008

[2] Deng C , Wu J, Cheng R, Meng F, Klok H-A, Zhong Z. Functional polypeptide and hybrid materials: precision synthesis via alpha-amino acid N-carboxyanhydride polymerization and emerging biomedical applications. Prog Polym Sci 2014;39:330–64.

[3] He X , Fan JW, Wooley KL. Stimuli-Triggered Sol–Gel transitions of polypeptides derived from -alpha-Amino acid N-Carboxyanhydride (NCA) polymerizations. Chem Asian J 2016;11:437–47. 26568257 10.1002/asia.201500957

[4] Deming TJ. Cobalt and iron initiators for the controlled polymerization of α-amino acid-*N*-carboxyanhydrides. Macromolecules 1999;32:4500–2.

[5] Deming TJ. Facile synthesis of block copolypeptides of defined architecture. Nature 1997;390:386–9. 9389476 10.1038/37084

[6] Dimitrov I , Schlaad H. Synthesis of nearly monodisperse polystyrene–polypeptide block copolymers via polymerisation of N-carboxyanhydrides. Chem Commun 2003;2944–5. 10.1039/b308990h 14680253

[7] Lu H , Cheng J. Hexamethyldisilazane-mediated controlled polymerization of α-amino acid *N*-carboxyanhydrides. J Am Chem Soc 2007;129:14114–5. 17963385 10.1021/ja074961q

[8] Lu H , Cheng J. *N*-trimethylsilyl amines for controlled ring-opening polymerization of amino acid *N*-carboxyanhydrides and facile end group functionalization of polypeptides. J Am Chem Soc 2008;130:12562–3. 18763770 10.1021/ja803304x

[9] Vayaboury W , Giani O, Cottet H, Deratani A, Schué F. Living polymerization of α-amino acid N-carboxyanhydrides(NCA) upon decreasing the reaction temperature. Macromol Rapid Commun 2004;25:1221–4.

[10] Aliferis T , Iatrou H, Hadjichristidis N. Living polypeptides. Biomacromolecules 2004;5:1653–6. 15360270 10.1021/bm0497217

[11] Zou J , Fan J, He X, Zhang S, Wang H, Wooley KL. A facile glovebox-free strategy to significantly accelerate the syntheses of well-defined polypeptides by *N*-carboxyanhydride (NCA) ring-opening polymerizations. Macromolecules 2013;46:4223–6. 23794753 10.1021/ma4007939 PMC3686519

[12] Li Y , Rodrigues J, Tomas H. Injectable and biodegradable hydrogels: gelation, biodegradation and biomedical applications. Chem Soc Rev 2012;41:2193–221. 22116474 10.1039/c1cs15203c

[13] Cao D , Ding J. Recent advances in regenerative biomaterials. Regen Biomater 2022;9:rbac098. 36518879 10.1093/rb/rbac098 PMC9745784

[14] Yeon B , Park MH, Moon HJ, Kim SJ, Cheon YW, Jeong B. 3D culture of adipose-tissue-derived stem cells mainly leads to chondrogenesis in poly(ethylene glycol)-poly(L-alanine) diblock copolymer thermogel. Biomacromolecules 2013;14:3256–66. 23909492 10.1021/bm400868j

[15] Yu L , Ding J. Injectable hydrogels as unique biomedical materials. Chem Soc Rev 2008;37:1473–81. 18648673 10.1039/b713009k

[16] Dou X-Q , Feng C-L. Amino acids and peptide-based supramolecular hydrogels for three-dimensional cell culture. Adv Mater 2017;29:1604062. 10.1002/adma.201604062 28112836

[17] Zhang S , Alvarez DJ, Sofroniew MV, Deming TJ. Design and synthesis of nonionic copolypeptide hydrogels with reversible thermoresponsive and tunable physical properties. Biomacromolecules 2015;16:1331–40. 25748800 10.1021/acs.biomac.5b00124 PMC5247266

[18] Choi YY , Jang JH, Park MH, Choi BG, Chi B, Jeong B. Block length affects secondary structure, nanoassembly and thermosensitivity of poly(ethylene glycol)-poly(l-alanine) block copolymers. J Mater Chem 2010;20:3416–21.

[19] Shinde UP , Joo MK, Moon HJ, Jeong B. Sol–gel transition of PEG–PAF aqueous solution and its application for hGH sustained release. J Mater Chem 2012;22:6072–9.

[20] Kang EY , Yeon B, Moon HJ, Jeong B. PEG-l-PAF and PEG-d-PAF: comparative study on thermogellation and biodegradation. Macromolecules 2012;45:2007–13.

[21] Cheng Y , He C, Xiao C, Ding J, Zhuang X, Huang Y, Chen X. Decisive role of hydrophobic side groups of polypeptides in thermosensitive gelation. Biomacromolecules 2012;13:2053–9. 22681239 10.1021/bm3004308

[22] Huang J , Hastings CL, Duffy GP, Kelly HM, Raeburn J, Adams DJ, Heise A. Supramolecular hydrogels with reverse thermal gelation properties from (oligo)tyrosine containing block copolymers. Biomacromolecules 2013;14:200–6. 23190093 10.1021/bm301629f

[23] He X , Fan J, Zhang F, Li R, Pollack KA, Raymond JE, Zou J, Wooley KL. Multi-responsive hydrogels derived from the self-assembly of tethered allyl-functionalized racemic oligopeptides. J Mater Chem B 2014;2:8123–30. 25485113 10.1039/C4TB00909F PMC4255538

[24] Xu Q , He C, Ren K, Xiao C, Chen X. Thermosensitive polypeptide hydrogels as a platform for ROS-Triggered cargo release with innate cytoprotective ability under oxidative stress. Adv Healthc Mater 2016;5:1979–90. 27283999 10.1002/adhm.201600292

[25] Song H , Yang G, Huang P, Kong D, Wang W. Self-assembled PEG–poly (l-valine) hydrogels as promising 3D cell culture scaffolds. J Mater Chem B 2017;5:1724–33. 32263913 10.1039/c6tb02969h

[26] Song H , Huang P, Niu J, Shi G, Zhang C, Kong D, Wang W. Injectable polypeptide hydrogel for dual-delivery of antigen and TLR3 agonist to modulate dendritic cells in vivo and enhance potent cytotoxic T-lymphocyte response against melanoma. Biomaterials 2018;159:119–29. 29324304 10.1016/j.biomaterials.2018.01.004

[27] Mai Y , Eisenberg A. Self-assembly of block copolymers. Chem Soc Rev 2012;41:5969–85. 22776960 10.1039/c2cs35115c

[28] Deming TJ. Synthetic polypeptides for biomedical applications. Prog Polym Sci 2007;32:858–75.

[29] Park MH , Joo MK, Choi BG, Jeong B. Biodegradable thermogels. Acc Chem Res 2012;45:424–33. 21992012 10.1021/ar200162j

[30] Nowak AP , Breedveld V, Pakstis L, Ozbas B, Pine DJ, Pochan D, Deming TJ. Rapidly recovering hydrogel scaffolds from self-assembling diblock copolypeptide amphiphiles. Nature 2002;417:424–8. 12024209 10.1038/417424a

[31] Breedveld V , Nowak AP, Sato J, Deming TJ, Pine DJ. Rheology of block copolypeptide solutions: hydrogels with tunable properties. Macromolecules 2004;37:3943–53.

[32] Yang CY , Song B, Ao Y, Nowak AP, Abelowitz RB, Korsak RA, Havton LA, Deming TJ, Sofroniew MV. Biocompatibility of amphiphilic diblock copolypeptide hydrogels in the central nervous system. Biomaterials 2009;30:2881–98. 19251318 10.1016/j.biomaterials.2009.01.056

[33] Popescu MT , Liontos G, Avgeropoulos A, Tsitsilianis C. Stimuli responsive fibrous hydrogels from hierarchical self-assembly of a triblock copolypeptide. Soft Matter 2015;11:331–42. 25379651 10.1039/c4sm02092h

[34] Hou S-S , Fan N-S, Tseng Y-C, Jan J-S. Self-assembly and hydrogelation of coil–sheet poly(l-lysine)-block-poly(l-threonine) block copolypeptides. Macromolecules 2018;51:8054–63.

[35] Pakstis LM , Ozbas B, Hales KD, Nowak AP, Deming TJ, Pochan D. Effect of chemistry and morphology on the biofunctionality of self-assembling diblock copolypeptide hydrogels. Biomacromolecules 2004;5:312–8. 15002989 10.1021/bm034249v

[36] Negri GE , Deming TJ. Triggered copolypeptide hydrogel degradation using photolabile lysine protecting groups. ACS Macro Lett 2016;5:1253–6. 35614735 10.1021/acsmacrolett.6b00715

[37] He C , Kim SW, Lee DS. In situ gelling stimuli-sensitive block copolymer hydrogels for drug delivery. J Control Release 2008;127:189–207. 18321604 10.1016/j.jconrel.2008.01.005

[38] Shi J , Yu L, Ding J. PEG-based thermosensitive and biodegradable hydrogels. Acta Biomater 2021;128:42–59. 33857694 10.1016/j.actbio.2021.04.009

[39] Branca C , Magazu S, Maisano G, Migliardo F, Migliardo P, Romeo G. Hydration study of PEG/water mixtures by quasi elastic light scattering, acoustic and rheological measurements. J Phys Chem B 2002;106:10272–6.

[40] Polik WF , Burchard W. Static Light-Scattering from aqueous poly(ethylene oxide) solutions in the temperature-range 20–90°C. Macromolecules 1983;16:978–82.

[41] Cui S , Yu L, Ding J. Semi-bald micelles and corresponding percolated micelle networks of thermogels. Macromolecules 2018;51:6405–20.

[42] Patel M , Park S, Lee HJ, Jeong B. Polypeptide thermogels as three-dimensional scaffolds for cells. Tissue Eng Regen Med 2018;15:521–30. 30603576 10.1007/s13770-018-0148-4 PMC6171707

[43] Choi YY , Joo MK, Sohn YS, Jeong B. Significance of secondary structure in nanostructure formation and thermosensitivity of polypeptide block copolymers. Soft Matter 2008;4:2383–7.

[44] Park SH , Choi BG, Moon HJ, Cho S-H, Jeong B. Block sequence affects thermosensitivity and nano-assembly: PEG-l-PA-dl-PA and PEG-dl-PA-l-PA block copolymers. Soft Matter 2011;7:6515–21.

[45] Jeong SY , Moon HJ, Park MH, Joo MK, Jeong B. Molecular captain: a light-sensitive linker molecule in poly(ethylene glycol)-poly(L-alanine)-poly(ethylene glycol) triblock copolymer directs molecular nano-assembly, conformation, and sol–gel transition. J Polym Sci A Polym Chem 2012;50:3184–91.

[46] Joo JH , Ko DY, Moon HJ, Shinde UP, Park MH, Jeong B. Ion and temperature sensitive polypeptide block copolymer. Biomacromolecules 2014;15:3664–70. 25178662 10.1021/bm500942p

[47] Thornton PD , Billah SMR, Cameron NR. Enzyme-degradable self-assembled hydrogels from Polyalanine-Modified poly(ethylene glycol) star polymers. Macromol Rapid Commun 2013;34:257–62. 23288556 10.1002/marc.201200649

[48] Jang JH , Choi YM, Choi YY, Joo MK, Park MH, Choi BG, Kang EY, Jeong B. pH/temperature sensitive chitosan-g-(PA-PEG) aqueous solutions as new thermogelling systems. J Mater Chem 2011;21:5484–91.

[49] Zhang Y , Song H, Zhang H, Huang P, Liu J, Chu L, Liu J, Wang W, Cheng Z, Kong D. Fine tuning the assembly and gel behaviors of PEGylated polypeptide conjugates by the copolymerization of l -alanine and γ-benzyl-l-glutamate *N* -carboxyanhydrides. J Polym Sci Part A Polym Chem 2017;55:1512–23.

[50] Han JO , Joo MK, Jang JH, Park MH, Jeong B. PVPylated poly(alanine) as a new thermogelling polymer. Macromolecules 2009;42:6710–5.

[51] Jeong Y , Joo MK, Bahk KH, Choi YY, Kim H-T, Kim W-K, Jeong Lee H, Sohn YS, Jeong B. Enzymatically degradable temperature-sensitive polypeptide as a new in-situ gelling biomaterial. J Control Release 2009;137:25–30. 19306901 10.1016/j.jconrel.2009.03.008

[52] Joo MK , Ko DY, Jeong SJ, Park MH, Shinde UP, Jeong B. Incorporation of d-alanine into poly(ethylene glycol) and l-poly(alanine-co-phenylalanine) block copolymers affects their nanoassemblies and enzymatic degradation. Soft Matter 2013;9:8014–22.

[53] Kang EY , Moon HJ, Joo MK, Jeong B. Thermogelling chitosan-*g*-(PAF-PEG) aqueous solution as an injectable scaffold. Biomacromolecules 2012;13:1750–7. 22607186 10.1021/bm300085c

[54] Oh HJ , Joo MK, Sohn YS, Jeong B. Secondary structure effect of polypeptide on reverse thermal gelation and degradation of L/DL-Poly(alanine)-Poloxamer-L/DL-Poly(alanine) copolymers. Macromolecules 2008;41:8204–9.

[55] Kim JY , Park MH, Joo MK, Lee SY, Jeong B. End groups adjusting the molecular nano-assembly pattern and thermal gelation of polypeptide block copolymer aqueous solution. Macromolecules 2009;42:3147–51.

[56] Choi BG , Park MH, Cho S-H, Joo MK, Oh HJ, Kim EH, Park K, Han DK, Jeong B. Thermal gelling polyalanine-poloxamine-polyalanine aqueous solution for chondrocytes 3D culture: initial concentration effect. Soft Matter 2011;7:456–62.

[57] Park MH , Choi BG, Jeong B. Complexation-Induced biomimetic long range fibrous orientation in a rigid-flexible block copolymer thermogel. Adv Funct Mater 2012;22:5118–25.

[58] Kim EH , Joo MK, Bahk KH, Park MH, Chi B, Lee YM, Jeong B. Reverse thermal gelation of PAF-PLX-PAF block copolymer aqueous solution. Biomacromolecules 2009;10:2476–81. 19637909 10.1021/bm9004436

[59] Moon HJ , Choi BG, Park MH, Joo MK, Jeong B. Enzymatically degradable thermogelling poly(alanine-co-leucine)-poloxamer-poly(alanine-co-leucine). Biomacromolecules 2011;12:1234–42. 21388161 10.1021/bm101518c

[60] Chen C , Wang Z, Li Z. Thermoresponsive polypeptides from pegylated poly-l-glutamates. Biomacromolecules 2011;12:2859–63. 21718026 10.1021/bm200849m

[61] Yu L , Fu W, Li Z. Tuning the phase transition temperature of thermal-responsive OEGylated poly- l -glutamate via random copolymerization with l-alanine. Soft Matter 2015;11:545–50. 25420954 10.1039/c4sm02270j

[62] Shen J , Chen C, Fu W, Shi L, Li Z. Conformation-specific self-assembly of thermo-responsive poly(ethylene glycol)-*b*-polypeptide diblock copolymer. Langmuir 2013;29:6271–8. 23634643 10.1021/la401095s

[63] Zhang S , Fu W, Li Z. Supramolecular hydrogels assembled from nonionic poly(ethylene glycol)-b-polypeptide diblocks containing OEGylated poly-l-glutamate. Polym Chem 2014;5:3346–51.

[64] Chen C , Wu D, Fu W, Li Z. Peptide hydrogels assembled from nonionic alkyl-polypeptide amphiphiles prepared by ring-opening polymerization. Biomacromolecules 2013;14:2494–8. 23822551 10.1021/bm4008259

[65] Shen Y , Zhang S, Wan Y, Fu W, Li Z. Hydrogels assembled from star-shaped polypeptides with a dendrimer as the core. Soft Matter 2015;11:2945–51. 25720319 10.1039/c5sm00083a

[66] Fu X , Shen Y, Ma Y, Fu W, Li Z. Tunable supramolecular hydrogels from polypeptide-PEG-polypeptide triblock copolymers. Sci China Chem 2015;58:1005–12.

[67] Zhao D , Li D, Quan F, Zhou Y, Zhang Z, Chen X, He C. Rapidly thermoreversible and biodegradable polypeptide hydrogels with sol–gel–sol transition dependent on subtle manipulation of side groups. Biomacromolecules 2021;22:3522–33. 34297548 10.1021/acs.biomac.1c00583

[68] Meng F , Ni Y, Ji S, Fu X, Wei Y, Sun J, Li Z. Dual thermal- and pH-responsive polypeptide-based hydrogels. Chin J Polym Sci 2017;35:1243–52.

[69] Liu D-L , Chang X, Dong C-M. Reduction- and thermo-sensitive star polypeptide micelles and hydrogels for on-demand drug delivery. Chem Commun (Camb) 2013;49:1229–31. 23288017 10.1039/c2cc38343h

[70] Gao Y , Dong C-M. Triple redox/temperature responsive diselenide-containing homopolypeptide micelles and supramolecular hydrogels thereof. J Polym Sci A Polym Chem 2018;56:1067–77.

[71] Ma Y , Fu X, Shen Y, Fu W, Li Z. Irreversible low critical solution temperature behaviors of thermal-responsive OEGylated poly (l-cysteine) containing disulfide bonds. Macromolecules 2014;47:4684–9.

[72] Turabee M , Thambi T, Duong HTT, Jeong JH, Lee DS. A pH- and temperature-responsive bioresorbable injectable hydrogel based on polypeptide block copolymers for the sustained delivery of proteins *in vivo*. Biomater Sci 2018;6:661–71. 29423489 10.1039/c7bm00980a

[73] Yu L , Zhang H, Ding JD. A subtle end-group effect on macroscopic physical gelation of triblock copolymer aqueous solutions. Angew Chem Int Ed Engl 2006;45:2232–5. 16518786 10.1002/anie.200503575

[74] Li D , Zhao D, He C, Chen X. Crucial impact of residue chirality on the gelation process and biodegradability of thermoresponsive polypeptide hydrogels. Biomacromolecules 2021;22:3992–4003. 34464095 10.1021/acs.biomac.1c00785

[75] Lee HJ , Jeong B. dl-Polyalanine as a PEG-Free thermogel. ACS Macro Lett 2022;11:825–9. 35686852 10.1021/acsmacrolett.2c00267

[76] Cheng Y , He C, Xiao C, Ding J, Cui H, Zhuang X, Chen X. Versatile biofunctionalization of polypeptide-based thermosensitive hydrogels via click chemistry. Biomacromolecules 2013;14:468–75. 23311471 10.1021/bm3017059

[77] Zhao D , Tang Q, Zhou Q, Peng K, Yang H, Zhang X. A photo-degradable injectable self-healing hydrogel based on star poly(ethylene glycol)-b-polypeptide as a potential pharmaceuticals delivery carrier. Soft Matter 2018;14:7420–8. 30187054 10.1039/c8sm01575a

[78] Lin Z , Ding J, Chen X, He C. pH‐ and temperature‐responsive hydrogels based on tertiary amine‐modified polypeptides for stimuli‐responsive drug delivery. Chem-Asian J 2023;18:e202300021. 36856525 10.1002/asia.202300021

[79] Shi Y , Li D, Ding J, He C, Chen X. Physiologically relevant pH- and temperature-responsive polypeptide hydrogels with adhesive properties. Polym Chem 2021;12:2832–9.

[80] Hamley IW , Cheng G, Castelletto V. A thermoresponsive hydrogel based on telechelic PEG End-Capped with hydrophobic dipeptides. Macromol Biosci 2011;11:1068–78. 21557478 10.1002/mabi.201100022

[81] Kirkham S , Castelletto V, Hamley IW, Reza M, Ruokolainen J, Hermida-Merino D, Bilalis P, Iatrou H. Self-assembly of telechelic tyrosine end-capped PEO and poly(alanine) polymers in aqueous solution. Biomacromolecules 2016;17:1186–97. 26867986 10.1021/acs.biomac.6b00023

[82] Sun Y , Hou Y, Zhou X, Yuan J, Wang J, Lu H. Controlled synthesis and enzyme-induced hydrogelation of poly (l-phosphotyrosine)s via ring-opening polymerization of α-amino acid *N* -carboxyanhydride. ACS Macro Lett 2015;4:1000–3. 35596435 10.1021/acsmacrolett.5b00429

[83] Liu R , Shi Z, Sun J, Li Z. Enzyme responsive supramolecular hydrogels assembled from nonionic peptide amphiphiles. Sci China Chem 2018;61:1314–9.

[84] He X , Fan J, Zou J, Wooley KL. Reversible photo-patterning of soft conductive materials via spatially-defined supramolecular assembly. Chem Commun (Camb) 2016;52:8455–8. 27305966 10.1039/c6cc03579e PMC5043637

[85] Zhao D , Zhou Q, Yang K, Yang H, Tang Q, Zhang X. An injectable ROS‐responsive self‐healing hydrogel based on tetra‐poly(ethylene glycol)‐b‐oligo (l‐methionine). Macromol Chem Phys 2019;220:1900106.

[86] Yu S , Wang C, Yu J, Wang J, Lu Y, Zhang Y, Zhang X, Hu Q, Sun W, He C, Chen X, Gu Z. Injectable bioresponsive gel depot for enhanced immune checkpoint blockade. Adv Mater 2018;30:1801527. 10.1002/adma.201801527 29786888

[87] Zhu H , Zhu M, Shuai S, Zhao C, Liu Y, Liu Y, Li X, Rao Z, Li Y, Hao J. Effect of polypeptide block length on nano-assembly morphology and thermo-sensitivity of methyl poly (ethylene glycol)-poly (L-valine) copolymer aqueous solutions. J Sol–Gel Sci Technol 2019;92:618–27.

[88] Ren X , Wang N, Zhou Y, Song A, Jin G, Li Z, Luan Y. An injectable hydrogel using an immunomodulating gelator for amplified tumor immunotherapy by blocking the arginase pathway. Acta Biomater 2021;124:179–90. 33524560 10.1016/j.actbio.2021.01.041

[89] Howard D , Buttery LD, Shakesheff KM, Roberts SJ. Tissue engineering: strategies, stem cells and scaffolds. J Anat 2008;213:66–72. 18422523 10.1111/j.1469-7580.2008.00878.x PMC2475566

[90] Lee KY , Mooney DJ. Hydrogels for tissue engineering. Chem Rev 2001;101:1869–79. 11710233 10.1021/cr000108x

[91] Khademhosseini A , Langer R. Microengineered hydrogels for tissue engineering. Biomaterials 2007;28:5087–92. 17707502 10.1016/j.biomaterials.2007.07.021

[92] Park MH , Moon HJ, Park JH, Shinde UP, Ko du Y, Jeong B. PEG-poly(L-alanine) thermogel as a 3D scaffold of bone-marrow-derived mesenchymal stem cells. Macromol Biosci 2015;15:464–72. 25515203 10.1002/mabi.201400426

[93] Peng S , Wu CW, Lin JY, Yang CY, Cheng MH, Chu IM. Promoting chondrocyte cell clustering through tuning of a poly(ethylene glycol)-poly(peptide) thermosensitive hydrogel with distinctive microarchitecture. Mater Sci Eng C Mater Biol Appl 2017;76:181–9. 28482515 10.1016/j.msec.2017.02.130

[94] Park J , Kim IY, Patel M, Moon HJ, Hwang S-J, Jeong B. 2D and 3D hybrid systems for enhancement of chondrogenic differentiation of Tonsil-Derived mesenchymal stem cells. Adv Funct Mater 2015;25:2573–82.

[95] Kye EJ , Kim SJ, Park MH, Moon HJ, Ryu KH, Jeong B. Differentiation of tonsil-tissue-derived mesenchymal stem cells controlled by surface-functionalized microspheres in PEG-polypeptide thermogels. Biomacromolecules 2014;15:2180–7. 24805903 10.1021/bm500342r

[96] Lee SS , Choi GE, Lee HJ, Kim Y, Choy JH, Jeong B. Layered double hydroxide and polypeptide thermogel nanocomposite system for chondrogenic differentiation of stem cells. ACS Appl Mater Interfaces 2017;9:42668–75. 29165981 10.1021/acsami.7b17173

[97] Choi BG , Park MH, Cho S-H, Joo MK, Oh HJ, Kim EH, Park K, Han DK, Jeong B. In situ thermal gelling polypeptide for chondrocytes 3D culture. Biomaterials 2010;31:9266–72. 20864172 10.1016/j.biomaterials.2010.08.067

[98] Liu H , Cheng Y, Chen J, Chang F, Wang J, Ding J, Chen X. Component effect of stem cell-loaded thermosensitive polypeptide hydrogels on cartilage repair. Acta Biomater 2018;73:103–11. 29684624 10.1016/j.actbio.2018.04.035

[99] Moon HJ , Patel M, Chung H, Jeong B. Nanocomposite versus mesocomposite for osteogenic differentiation of tonsil-derived mesenchymal stem cells. Adv Healthc Mater 2016;5:353–63. 26634888 10.1002/adhm.201500558

[100] Huang W-S , Chu I-M. Injectable polypeptide hydrogel/inorganic nanoparticle composites for bone tissue engineering. PLoS One 2019;14:e0210285. 30629660 10.1371/journal.pone.0210285 PMC6328128

[101] Zhang S , Burda JE, Anderson MA, Zhao Z, Ao Y, Cheng Y, Sun Y, Deming TJ, Sofroniew MV. Thermoresponsive copolypeptide hydrogel vehicles for central nervous system cell delivery. ACS Biomater Sci Eng 2015;1:705–17. 27547820 10.1021/acsbiomaterials.5b00153 PMC4991036

[102] Patel M , Moon HJ, Jung BK, Jeong B. Microsphere-Incorporated hybrid thermogel for neuronal differentiation of tonsil derived mesenchymal stem cells. Adv Healthc Mater 2015;4:1565–74. 26033880 10.1002/adhm.201500224

[103] Yun EJ , Yon B, Joo MK, Jeong B. Cell therapy for skin wound using fibroblast encapsulated poly(ethylene glycol)-poly(L-alanine) thermogel. Biomacromolecules 2012;13:1106–11. 22394182 10.1021/bm2018596

[104] Hong JH , Lee HJ, Jeong B. Injectable polypeptide thermogel as a tissue engineering system for hepatogenic differentiation of tonsil-derived mesenchymal stem cells. ACS Appl Mater Interfaces 2017;9:11568–76. 28290667 10.1021/acsami.7b02488

[105] Moon HJ , Lee HJ, Patel M, Park S, Chang SH, Jeong B. Hepatogenic supported differentiation of mesenchymal stem cells in a lactobionic acid-conjugated thermogel. ACS Macro Lett 2017;6:1305–9. 35650787 10.1021/acsmacrolett.7b00802

[106] Park MH , Yu Y, Moon HJ, Ko du Y, Kim HS, Lee H, Ryu KH, Jeong B. 3D culture of tonsil-derived mesenchymal stem cells in poly(ethylene glycol)-poly(L-alanine-co-L-phenyl alanine) thermogel. Adv Healthc Mater 2014;3:1782–91. 24958187 10.1002/adhm.201400140

[107] Kim H , Woo Y, Patel M, Jeong B. Thermogelling inclusion complex system for Fine-Tuned osteochondral differentiation of mesenchymal stem cells. Biomacromolecules 2020;21:3176–85. 32640158 10.1021/acs.biomac.0c00623

[108] Deng J , Wang X, Zhang W, Sun L, Han X, Tong X, Yu L, Ding J, Yu L, Liu Y. Versatile hypoxic extracellular vesicles laden in an injectable and bioactive hydrogel for accelerated bone regeneration. Adv Funct Mater 2023;33:2211664.

[109] Song B , Song J, Zhang S, Anderson MA, Ao Y, Yang CY, Deming TJ, Sofroniew MV. Sustained local delivery of bioactive nerve growth factor in the Central nervous system via tunable diblock copolypeptide hydrogel depots. Biomaterials 2012;33:9105–16. 22985994 10.1016/j.biomaterials.2012.08.060

[110] Zhang S , Anderson MA, Ao Y, Khakh BS, Fan J, Deming TJ, Sofroniew MV. Tunable diblock copolypeptide hydrogel depots for local delivery of hydrophobic molecules in healthy and injured central nervous system. Biomaterials 2014;35:1989–2000. 24314556 10.1016/j.biomaterials.2013.11.005 PMC3984939

[111] Wollenberg AL , O'Shea TM, Kim JH, Czechanski A, Reinholdt LG, Sofroniew MV, Deming TJ. Injectable polypeptide hydrogels via methionine modification for neural stem cell delivery. Biomaterials 2018;178:527–45. 29657091 10.1016/j.biomaterials.2018.03.057 PMC6054810

[112] Lin H-C , Chen C-Y, Kao C-W, Wu S-T, Chen C-L, Shen C-R, Juang J-H, Chu IM. In situ gelling-polypeptide hydrogel systems for the subcutaneous transplantation of MIN6 cells. J Polym Res 2020;27:64.

[113] Ma H , He C, Chen X. Injectable hydrogels as local depots at tumor sites for antitumor immunotherapy and immune‐based combination therapy. Macromol Biosci 2021;21:2100039. 10.1002/mabi.202100039 33818918

[114] Bai Y , Wang T, Zhang S, Chen X, He C. Recent advances in organic and polymeric carriers for local tumor chemo-immunotherapy. Sci China Technol Sci 2022;65:1011–28.

[115] Cheng Y , He C, Ding J, Xiao C, Zhuang X, Chen X. Thermosensitive hydrogels based on polypeptides for localized and sustained delivery of anticancer drugs. Biomaterials 2013;34:10338–47. 24095250 10.1016/j.biomaterials.2013.09.064

[116] Lv Q , He C, Quan F, Yu S, Chen X. DOX/IL-2/IFN-gamma co-loaded thermo-sensitive polypeptide hydrogel for efficient melanoma treatment. Bioact Mater 2018;3:118–28. 29744449 10.1016/j.bioactmat.2017.08.003 PMC5935762

[117] Cao D , Guo W, Cai C, Tang J, Rao W, Wang Y, Wang Y, Yu L, Ding J. Unified therapeutic‐prophylactic vaccine demonstrated with a postoperative filler gel to prevent tumor recurrence and metastasis. Adv Funct Mater 2022;32:226084.

[118] Wu X , Wu Y, Ye H, Yu S, He C, Chen X. Interleukin-15 and cisplatin co-encapsulated thermosensitive polypeptide hydrogels for combined immuno-chemotherapy. J Control Release 2017;255:81–93. 28408199 10.1016/j.jconrel.2017.04.011

[119] Yu S , Wei S, Liu L, Qi D, Wang J, Chen G, He W, He C, Chen X, Gu Z. Enhanced local cancer therapy using a CA4P and CDDP co-loaded polypeptide gel depot. Biomater Sci 2019;7:860–6. 30698593 10.1039/c8bm01442f

[120] Ding J , Wang T, Chen Z, Lin Z, Chen X, He C. Enhanced antitumor chemo‐immunotherapy by local co‐delivery of chemotherapeutics, immune checkpoint blocking antibody and IDO inhibitor using an injectable polypeptide hydrogel. J Polym Sci 2022;60:1595–609.

[121] Chen Z , Rong Y, Ding J, Cheng X, Chen X, He C. Injectable polypeptide hydrogel depots containing dual immune checkpoint inhibitors and doxorubicin for improved tumor immunotherapy and Post-Surgical tumor treatment. Pharmaceutics 2023;15:428. 36839750 10.3390/pharmaceutics15020428 PMC9965187

[122] Shinde UP , Moon HJ, Ko DY, Jung BK, Jeong B. Control of rhGH release profile from PEG-PAF thermogel. Biomacromolecules 2015;16:1461–9. 25849077 10.1021/acs.biomac.5b00325

[123] Shen W , Luan J, Cao L, Sun J, Yu L, Ding J. Thermogelling polymer-platinum(IV) conjugates for long-term delivery of cisplatin. Biomacromolecules 2015;16:105–15. 25435165 10.1021/bm501220a

[124] Yu S , Zhang D, He C, Sun W, Cao R, Cui S, Deng M, Gu Z, Chen X. Injectable thermosensitive polypeptide-based CDDP-complexed hydrogel for improving localized antitumor efficacy. Biomacromolecules 2017;18:4341–8. 29141405 10.1021/acs.biomac.7b01374

[125] Yu S , He C, Chen X. Injectable hydrogels as unique platforms for local Chemotherapeutics-Based combination antitumor therapy. Macromol Biosci 2018;18:e1800240. 30303620 10.1002/mabi.201800240

[126] Lv Q , Yu S, Quan F, He C, Chen X. Thermosensitive polypeptide hydrogels Co‐loaded with two anti‐tumor agents to reduce multi‐drug resistance and enhance local tumor treatment. Adv Therap 2020;3:1900165.

[127] Zheng Y , Cheng Y, Chen J, Ding J, Li M, Li C, Wang J, Chen X. Injectable Hydrogel-Microsphere construct with sequential degradation for locally synergistic chemotherapy. ACS Appl Mater Interfaces 2017;9:3487–96. 28067493 10.1021/acsami.6b15245

[128] Commins SP , Borish L, Steinke JW. Immunologic messenger molecules: cytokines, interferons, and chemokines. J Allergy Clin Immunol 2010;125:S53–72. 19932918 10.1016/j.jaci.2009.07.008

[129] Wu X , He C, Wu Y, Chen X, Cheng J. Nanogel-incorporated physical and chemical hybrid gels for highly effective chemo-protein combination therapy. Adv Funct Mater 2015;25:6744–55.

[130] Song H , Yang P, Huang P, Zhang C, Kong D, Wang W. Injectable polypeptide hydrogel-based co-delivery of vaccine and immune checkpoint inhibitors improves tumor immunotherapy. Theranostics 2019;9:2299–314. 31149045 10.7150/thno.30577 PMC6531311

[131] Turabee MH , Thambi T, Lym JS, Lee DS. Bioresorbable polypeptide-based comb-polymers efficiently improves the stability and pharmacokinetics of proteins in vivo. Biomater Sci 2017;5:837–48. 28287223 10.1039/c7bm00128b

[132] Bevilacqua MP , Huang DJ, Wall BD, Lane SJ, Edwards CK, Hanson JA, Benitez D, Solomkin JS, Deming TJ. Amino acid block copolymers with broad antimicrobial activity and barrier properties. Macromol Biosci 2017;17:1600492. 10.1002/mabi.201600492 28248002

[133] Cabral H , Kataoka K. Progress of drug-loaded polymeric micelles into clinical studies. J Control Release 2014;190:465–76. 24993430 10.1016/j.jconrel.2014.06.042

[134] Tang Z , He C, Tian H, Ding J, Hsiao BS, Chu B, Chen X. Polymeric nanostructured materials for biomedical applications. Prog Polym Sci 2016;60:86–128.

